# Microbial biotherapeutic metabolite alleviates liver injury by restoring hepatic lipid metabolism through PPARα across the gut-liver axis

**DOI:** 10.1128/mbio.01718-25

**Published:** 2025-08-12

**Authors:** Dylan J. Kramer, Weicang Wang, Ikaika Loque, Chara J. Walters-Laird, Christophe Morisseau, Xiaoyue Xiao, Marie Nearing, Clarissa Santos Rocha, Abhaya Dandekar, Bruce Hammock, Satya Dandekar

**Affiliations:** 1 Department of Medical Microbiology and Immunology, School of Medicine, University of California Davis 12218, Davis, California, USA; 2 Department of Entomology and Nematology, University of California Davis 8789, Davis, California, USA; 3 Department of Plant Sciences, College of Agriculture and Environmental Sciences, University of California Davis 117143, Davis, California, USA; University of Hawaii at Manoa, Honolulu, Hawaii, USA

**Keywords:** NAFLD, gut-liver axis, microbial metabolite, biotherapeutic, 10-hydroxystearic acid

## Abstract

Infectious and non-infectious liver diseases are marked by disrupted liver metabolism and are frequently accompanied by gut epithelial barrier dysfunction and microbial dysbiosis, reflecting the compromised gut-liver axis. Despite the pivotal role of the gut-liver axis in health, transformative therapeutic interventions that simultaneously target both the liver and gut remain underexplored. Peroxisome proliferator-activated receptor alpha (PPARα) suppression drives both gut and liver metabolic diseases. In this study, we report on the therapeutic impact of microbial metabolite, 10-hydroxystearic acid (10-HSA), on restoring lipid metabolism and liver regeneration through PPARα activation, leading to a functional gut-liver axis in an *in vivo* liver injury model. We previously identified 10-HSA, a known PPARα agonist, in *Lactiplantibacillus plantarum-*treated intestine. Here, we report that oral administration of 10-HSA prevented AFB1-induced gut epithelial barrier disruption and preserved mucosal T cell populations. Prominent downstream effects of 10-HSA-activated PPARα signaling included significant upregulation of known PPARα-regulated gene expression in the gut and liver; prevention of fibrotic changes and reduction of TGF-β signaling-related gene expression in the gut and liver; and activation of toxicant clearance metabolic pathways in the liver through the PPARα-NRF2 pathway. Restoration of the functional gut microenvironment during 10-HSA treatment was evident by increased gut microbial diversity and circulating citrulline levels. Our findings unveil a novel therapeutic trajectory that harnesses a single microbial metabolite to activate PPARα-mediated tissue repair/renewal pathways across the gut-liver axis, offering a promising biologic therapeutic for treatment of metabolic and inflammatory liver diseases.

**IMPORTANCE:**

Chronic liver diseases, including liver steatosis and fibrosis, are driven in part by dysregulation of PPARα and lipid metabolism. These diseases also generate gut barrier disruption and microbiome dysbiosis, leading to dysfunction of the gut-liver axis. Therapeutic strategies that concurrently support liver regeneration and gut mucosal repair can be highly effective in resolving liver metabolic diseases but remain underexplored. Microbial biotherapeutic metabolite 10-HSA induced repair and regeneration of both liver and gut through the activation of PPARα and restored lipid metabolism. Our findings reveal the therapeutic potential of a single microbial bioactive lipid molecule to repair both hepatic and gut mucosal sites simultaneously with important ramifications for treatment of diseases that disrupt the gut-liver axis.

## INTRODUCTION

Liver disease accounts for an estimated 4% of global deaths, while non-alcoholic fatty liver disease (NAFLD) affects approximately 32% of the global population (1). Infectious and non-infectious origins of NAFLD involve distinct mechanisms but share similar pathologies, including lipid metabolism dysregulation and impaired liver regeneration (2–4). Gut microbiome dysbiosis and disruption of epithelial barriers are frequently associated with liver diseases, which exacerbate liver inflammation and accelerate disease progression (5). These underscore the importance of maintaining gut-liver axis homeostasis and highlight the need for developing therapeutic strategies that can simultaneously repair and renew both the gut and liver for effective management of liver diseases.

Aflatoxin-β1 (AFB1) is a hepatotoxic and carcinogenic mycotoxin produced by fungi in the genus *Aspergillus* and provides an established model of liver injury. Dietary exposure of AFB1 through the consumption of contaminated foods causes liver damage, leading to NAFLD pathology, reflecting disease processes of both infectious and non-infectious liver-damaging agents (6–8). An estimated five billion people, mostly in developing countries in the world, are at risk of aflatoxin exposure (9). Aflatoxicosis is associated with high mortality in at-risk populations in these regions and contributes to cirrhosis, cholestasis, oxidative stress, and toxicant-associated fatty liver disease (TAFLD) progression, in part by disrupting lipid metabolism (9–12). Importantly, AFB1 exposure disrupts the gut-liver axis, resulting in damaged gut epithelial barriers and suppression of mucosal immunity (7, 13, 14). This is partly driven by increased immunosuppressive and pro-fibrotic TGF-β signaling, which plays a central role in NAFLD pathogenesis (15). Due to the remarkable similarities between AFB1-induced liver disease and other NAFLDs, murine models of AFB1 exposure provide an excellent opportunity to investigate novel therapeutic candidates to restore the gut-liver axis and resolve liver diseases.

Peroxisome proliferator-activated receptor alpha (PPARα) is a known metabolic regulator of lipid metabolism in the gut and liver and is an attractive therapeutic target for metabolic syndromes and NAFLD (16, 17). PPARα activity is known to be suppressed in fatty liver diseases (18). PPARα transcriptional activation and associated NF-κB inhibition have been shown to provide liver protection (19). Notably, PPARα can inhibit TGF-β production and signaling through suppression of NF-κB and SMAD2 activity (20, 21). Persistent TGF-β signaling promotes collagen deposition and fibrosis, which is a driving factor of cirrhosis in NAFLD (22–24). Beyond inhibiting TGF-β production, activation of PPARα restores fatty acid metabolism (FAM). Dysregulated FAM is a key driver of NAFLD. We previously reported that repair and regeneration of the damaged gut epithelial barrier are mediated through PPARα activation (16). Collectively, PPARα plays a critical role in hepatic and gut homeostasis. We hypothesize that activation of PPARα can be therapeutically leveraged to overcome gut-liver axis dysregulation for resolution of liver disease.

 Microbiome-based therapies have been explored for managing liver and gastrointestinal diseases (25). However, clinical studies using probiotics to restore the gut microbiome have yielded inconsistent results in reducing gut mucosal damage and preventing disease pathogenesis (26, 27). An alternative approach is being explored to use microbial metabolites to recapitulate the therapeutic effects of probiotics. Currently, the therapeutic potential of microbially derived biotherapeutics for restoring the gut-liver axis in liver disease is underexplored. We previously reported the rapid repair of gut epithelial barrier damage through PPARα activation following *Lactiplantibacillus plantarum* (LP) administration (16). We identified a microbial metabolite, known PPARα agonist 10-hydroxystearic acid (10-HSA), from the LP-treated gut (28). In the current study, we report that the microbial fatty acid biotherapeutic metabolite 10-HSA prevented AFB1-associated liver and gut mucosal dysfunction. This protective effect is mediated through PPARα activation and its downstream effects on the restoration of lipid energy metabolism and suppression of TGF-β signaling. Our findings highlight the innovative therapeutic opportunity to repair and renew both the liver and gut mucosa simultaneously in NAFLD and related metabolic liver diseases. 

## RESULTS

### Restoration of hepatic energy metabolism through PPARα activation

**Fig 1:**
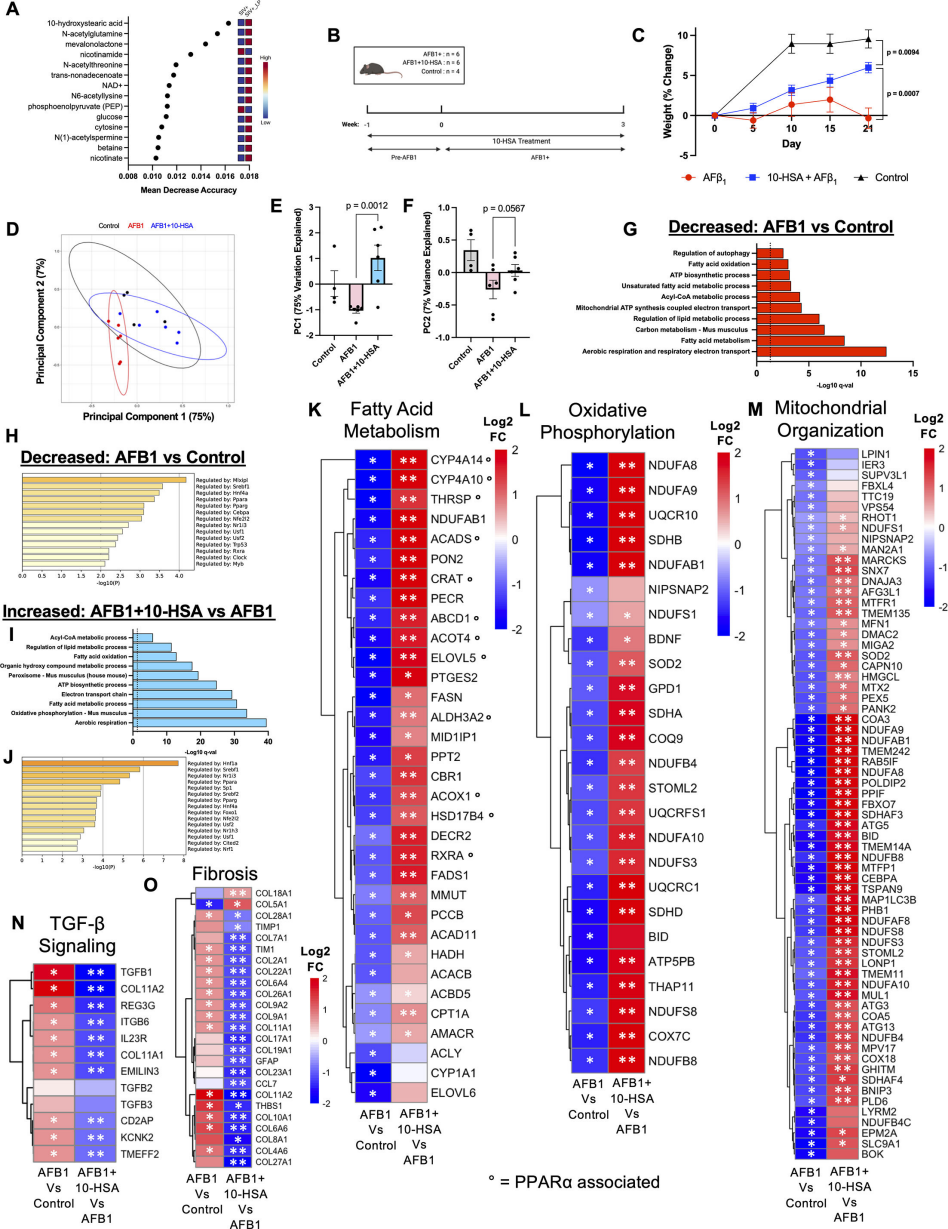
Restoration of liver energy metabolism. (**A**) Random forest mean decrease accuracy analysis of metabolites between SIV+LP and SIV+ gut luminal contents. (**B**) Experimental design and timeline of AFB1 exposure and 10-HSA treatment in mice. (**C**) Percent weight change during treatments. (**D**) PCA plot of liver RNA-seq data. Isolation of (**E**) PC1 and (**F**) PC2 to show separation between experimental groups with statistical analysis. (**G**) Decreased expression of sub-pathways in AFB1-exposed mice compared to healthy controls. (**H**) Decreased transcription factor activity, as determined by TRRUST analysis in AFB1-exposed mice compared to healthy controls. (**I**) Increased expression of sub-pathways in AFB1 + 10-HSA mice compared to AFB1-only animals. (**J**) Increased transcription factor activity, as determined by TRRUST analysis in AFB1 + 10-HSA mice compared to AFB1-only animals. Heatmaps showing genes within (**K**) fatty acid metabolism, (**L**) oxidative phosphorylation, (**M**) mitochondrial organization, (**N**) TGF-β production, and (**O**) fibrosis pathways from liver RNA-seq data. Data in bar charts represent mean and standard error. (*, FDR < 0.05; **, FDR < 0.01).

Our previous study demonstrated the therapeutic capacity of LP to repair damaged gut epithelial barriers in the virally inflamed and disrupted gut in the nonhuman primate model of HIV/AIDS (16). Untargeted metabolomic analysis of the gut luminal contents from *in vivo* LP-treated and untreated SIV-infected intestinal tissues identified production of 10-HSA, a PPARα agonist. Using random forest analysis, 10-HSA was shown to be the most important metabolite within the metabolomic signatures to discriminate and distinguish LP-treated versus untreated SIV+ intestine (Fig. 1A) (28). 10-HSA had the highest mean decrease accuracy score, indicating it as a highly influential metabolite, potentially accounting for the biotherapeutic effects of LP (Fig. 1A). We sought to determine the effect of 10-HSA as a PPARα activator on counteracting AFB1-induced NAFLD and for restoring the gut-liver axis function. Oral treatment of 10-HSA (100 mg/kg/daily) in mice was initiated 1 week prior to AFB1 exposure in drinking water (5 mg/L), and daily treatment was continued through the course of the study (Fig. 1B). Animals exposed to AFB1 showed failure to steadily gain weight during the study, while the unexposed healthy controls (control) showed a steady weight gain with an approximate 10% increase in body weight (Fig. 1C). Animals receiving 10-HSA during AFB1 exposure (AFB1 + 10-HSA) showed significant improvement in weight gain compared to animals with AFB1 exposure only (Fig. 1C).

Appearance of symptoms characteristic of NAFLD liver steatosis and fibrosis are well documented during AFB1 exposure (11, 29). However, the underlying mechanisms and transcriptomic signatures driving liver metabolic dysfunction during AFB1 exposure are under-investigated. We sought to determine the primarily molecular mechanisms driving dysregulation of FAM in the liver through gene expression analysis using RNA-seq. The PCA plot showed a marked separation among the AFB1-exposed, 10-HSA-treated AFB1-exposed, and untreated healthy control groups (Fig. 1D). AFB1 + 10-HSA-treated animals showed significant separation from AFB1 animals on PC1, which explained 75% of the variance in the data (Fig. 1E). PC2 also showed notable separation between AFB1 + 10-HSA and AFB1 treatment groups (Fig. 1F). Changes in liver gene expression were striking and significant in response to AFB1 exposure and 10-HSA treatment. We utilized FDR < 0.05 to determine significance for pathway analysis. Functional pathways pertaining to mitochondrial organization, monocarboxylic metabolism, and amino acid metabolism showed significant (*q* < 0.05) suppression in AFB1-exposed mice compared to untreated healthy control animals (Fig. S1A). We sought to understand the impact of AFB1 exposure on liver energy metabolism by interrogating significantly (*q* < 0.05) altered sub-pathways that underlie the main functional pathways related to energy generation or lipid metabolism. This in-depth analysis revealed highly significant downregulation of ten critical pathways related to cellular energy generation following AFB1 exposure (Fig. 1G). Gene expression regulating FAM and related pathways showed remarkable suppression with AFB1 exposure (Fig. 1G). In addition, pathways of mitochondrial electron transport chain (ETC) and ATP biosynthesis were downmodulated (Fig. 1G). As a primary site of FAM, the liver relies heavily on PPARα-regulated gene transcription to generate ATP from dietary fats (30). Through TRRUST analysis of significantly downregulated genes, a significant decrease in PPARα regulated gene expression was detected in the AFB1-exposed liver (Fig. 1H).

 The 10-HSA treatment resulted in a profound rescue of gene expression regulating translation, aerobic respiration and ETC, mitochondrial organization, and lipid metabolism in AFB1-exposed mice (Fig. S1B and C). Additionally, analysis of sub-pathways relating to energy metabolism revealed massive and significant increases in gene expression regulating aerobic respiration and FAM processes (Fig. 1I). The 10-HSA treatment significantly counteracted the AFB1 exposure-induced decrease in the expression of FAM, ETC, mitochondrial organization, ATP synthesis, and acyl-CoA metabolism-related pathways (Fig. 1I). These findings demonstrated a remarkable beneficial impact on liver function due to oral administration of 10-HSA. TRRUST analysis revealed PPARα-related gene expression was significantly upregulated due to 10-HSA treatment in the liver of AFB1-exposed animals (Fig. 1J). In addition, 10-HSA treatment led to increased transcriptional activity of PPARγ, SREBF1, HFN4A, NFE2L2 (NRF2), USF1, and USF2, all of which were significantly downregulated in AFB1-exposed mice (Fig. 1I and J). These transcription factors are major regulators of lipid and energy metabolism and oxidative stress management (31–34). Furthermore, FOXO1 showed upregulated transcriptional activity in 10-HSA-treated mice (Fig. 1J). This transcription factor is known to protect organisms from the impact of oxidative stress, a key driver of AFB1 pathogenesis (35). Increased activity of NRF1 and NRF2 was noted with 10-HSA treatment, which are critical drivers of mitochondrial biogenesis (Fig. 1J) (36).

 Side-by-side evaluation of significantly downregulated genes in AFB1 vs untreated controls as well as AFB1 + 10-HSA vs AFB1 only revealed striking changes in the transcriptional response to individual and combination treatments. Critical genes involved in FAM *ACADS, ABCD1, ACOT4, ACOX1,* and *CPT1A*, all of which are regulated by PPARα, showed a significant decrease in AFB1-exposed mice compared to unexposed controls (Fig. 1K). 10-HSA treatment was able to restore the expression of all five genes through the activation of PPARα (Fig. 1J and K). Oxidative phosphorylation genes, especially those encoding subunits of complex I of the ETC (NADH dehydrogenase), showed significantly decreased expression in AFB1-exposed mouse livers (Fig. 1L). This indicates that AFB1 not only disrupts FAM but also limits the capacity of proper ETC performance, which could dramatically reduce the capabilities of generating ATP. Genes encoding subunits of both complex IV (cytochrome C oxidase) and V (ATP synthase) showed significantly reduced expression upon AFB1 exposure (Fig. 1L). The 10-HSA treatment led to a remarkable increase in the expression of these critical genes in the liver of AFB1-exposed mice (Fig. 1L).

We previously showed that LP treatment induced recovery of mitochondrial organization and morphology *in vivo* in the nonhuman primate model of HIV/AIDS (16). The production of 10-HSA, a known PPARα agonist, was uniquely detected in gut luminal contents with LP treatment (Fig. 1A). RNA-seq data of AFB1-exposed mice treated with 10-HSA showed a dramatic increase in genes regulating mitochondrial organization (Fig. 1M). This included the upregulation of gene expression of mitochondrial transmembrane proteins *TMEM135, TMEM242, TMEM14A,* and *TMEM11* (Fig. 1M). Expression of several genes encoding subunits of complex I was increased in 10-HSA-treated mice (Fig. 1M).

Compared to healthy control animals, TGF-β production and signaling-related gene expression was increased in AFB1-exposed animals (Fig. 1K; Fig. S2A). The data indicated that AFB1 elicits TGF-β production in the liver, which we hypothesized drives immunosuppression across the gut-liver axis. *TGFB1*, which encodes TGF-β, showed significantly reduced expression in the liver of AFB1 + 10-HSA treated compared to AFB1-only animals (Fig. 1N). AFB1 significantly increased the expression of *TGFB1* compared to control animals (Fig. 1N). PPARα signaling is known to decrease TGF-β production and signaling; thereby we hypothesize that decreased TGFB1 transcription with 10-HSA treatment is a PPARα-mediated mechanism (37). We observed that this 10-HSA-driven decrease in TGF-β led to a significant decrease in the expression of 18 collagen genes (COL, Fig. 1O). Many of these significantly decreased collagen genes were remarkably upregulated in AFB1-exposed mice, indicative of increased hepatic fibrosis. In addition, genes *GFAP, TIM1, TIMP1, *and* THBS1* showed significant downmodulation with 10-HSA treatment (Fig. 1O). All these genes are involved in promoting fibrotic changes, especially *GFAP*, *TIMP1,* and *THBS1,* which are expressed by activated hepatic stellate cells, thereby driving liver fibrosis (38–40).

Aflatoxin exposure is known to dampen cellular autophagy functions and enhance apoptosis instead (41). Lack of autophagy has been associated with AFB1-induced liver toxicity (42). In AFB1-exposed mice, regulation of autophagy, a sub-pathway of cellular catabolism, showed significant (*q* = 0.003) reduction (Fig. S2B). The 10-HSA-treated mice showed increased expression of many genes within the autophagy pathway, which were significantly suppressed in AFB1-exposed mice (Fig. S2B). Increased expression of autophagy-related genes by 10-HSA could lead to decreased hepatocyte apoptosis, thereby salvaging liver function (43). PPARα activation has been shown to promote autophagy pathways (37).

During 10-HSA treatment, the most upregulated pathway was of transcription (*q* = 1e−49), with a significant increase in 127 genes that were mapped to this pathway (Fig. S1B). Many ribosomal protein (RPL)-coding genes were significantly upregulated in AFB1 + 10-HSA treatment compared to AFB1-exposed mice (Fig. S1C). Importantly, many mitochondrial specific ribosomal proteins (MRPL) also showed significantly increased expression with 10-HSA treatment (Fig. S1C).

A significant downregulation of genes involved in DNA damage response and DNA metabolism-related processes was detected in 10-HSA-treated animals compared to AFB1-only exposed animals, indicative of enhanced AFB1 detoxification (Fig. S2C). Therefore, 10-HSA treatment reduced the need for cellular machinery required for DNA repair by mitigating AFB1-induced damage. Collectively, these data demonstrate the dramatic impact of 10-HSA on promoting PPARα-mediated energy metabolism-related gene expression, mitochondrial organization, and reducing TGF-β signaling in the liver. Therefore, 10-HSA treatment counteracts dysregulated pathways in the liver, which are known to promote steatosis and fibrosis in the liver.

### Activation of AFB1 detoxifying pathways in the liver

**Fig 2:**
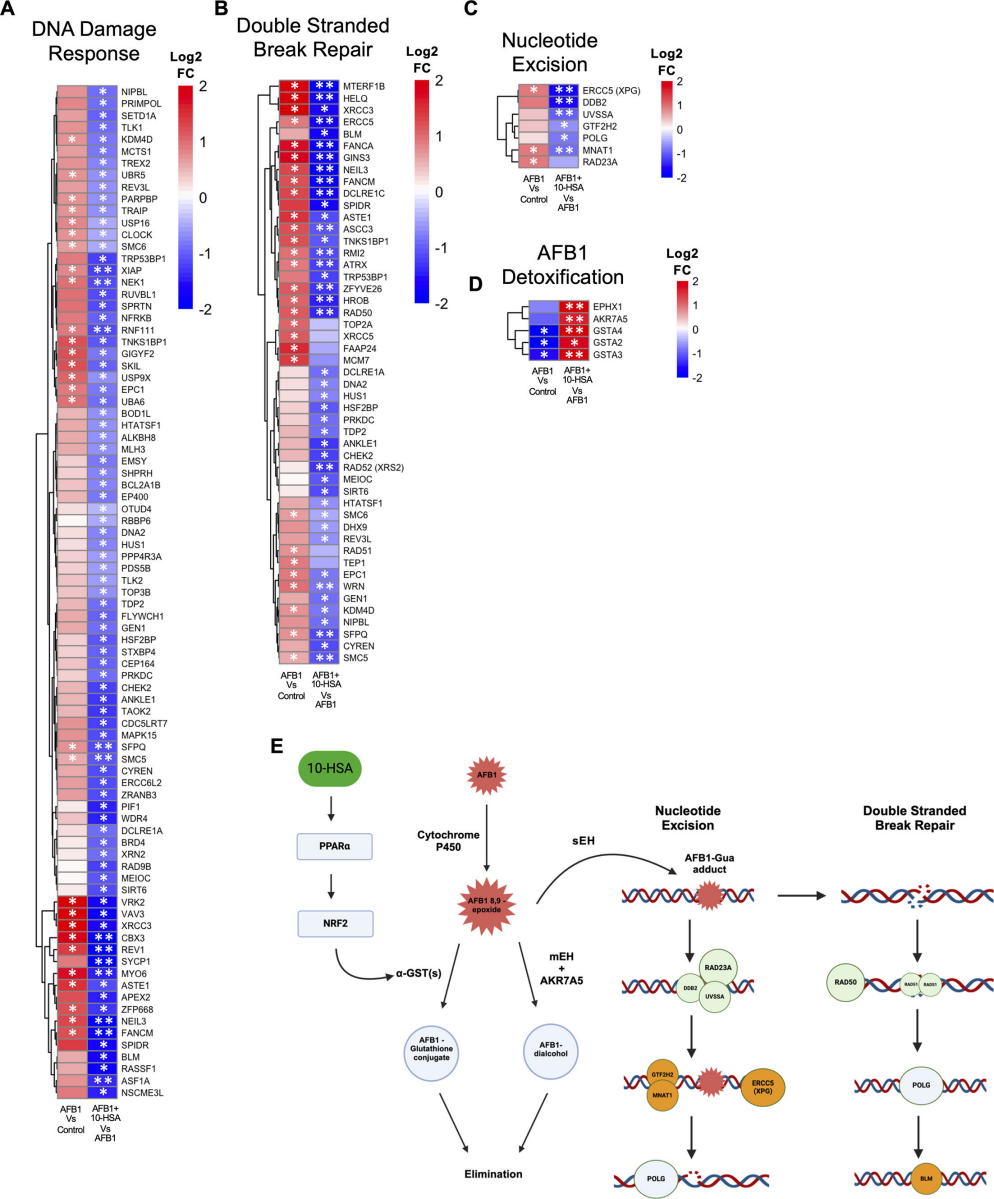
Detoxification of AFB1 in the liver through PPARα-NRF2 signaling. RNA-seq data from the liver showing (**A**) DNA damage response, (**B**) double-stranded break repair, (**C**) nucleotide excision, and (**D**) AFB1 detoxification pathways. (**E**) Proposed pathway for activation of AFB1 detoxification by 10-HSA.

** **Transcriptomic analysis revealed significant reduction in the DNA-repair pathway in 10-HSA-treated animals compared to AFB1-only exposed animals (Fig. S2C). AFB1 is converted into its 8,9-epoxide form through cytochrome p450 (CYP450) enzymatic activity, whereby aflatoxin-DNA adducts with guanine nucleotides are formed (44–46). These adducts drive the carcinogenic changes commonly reported with AFB1 exposure. Nucleotide excision and DNA repair processes are required to reduce aflatoxin-DNA adducts. AFB1-exposed animals showed significant upregulation of genes involved in DNA damage response, double-stranded break repair, and nucleotide excision (Fig. 2A through C). Importantly, *RAD51* was significantly upregulated in AFB1 animals (Fig. 2B). This gene encodes a protein critical for formation of RAD51 fibrils, which aid in the reconnection of a double-stranded break, a characteristic aspect of aflatoxin-DNA adduct formation (47). Another gene *ERCC5(XPG*) showed significantly increased expression with AFB1-exposed animals (Fig. 2C). XPG is a critical protein for nucleotide excision and operates by cleaving DNA at the necessary site and recruiting proteins to unwind and facilitate nucleotide excision (48). AFB1 exposure significantly upregulated *NEIL3* expression, a gene crucial for oxidized nucleotide excision (Fig. 2A) (49). The 10-HSA treatment led to significant reduction of DNA-repair requirements in the AFB1-exposed liver (Fig. 2A through C), as seen by significant downregulation of *ERCC5 (XPG*) and of *BLM*, *GTF2H2*, *NEIL3,* and *DDB2* gene expressions. Decreased expression of these genes indicates that nucleotide excision and DNA damage repair requirements in the liver were reduced in response to 10-HSA treatment.

To determine the mechanism underlying decreased DNA damage during 10-HSA treatment, we searched for known AFB1 detoxifying enzymes in the transcriptomic data set. Detoxification of AFB1 is well documented through glutathione S-transferase (GST) and aldo-keto reductase (AKR) activity (46). A significant increase in three alpha GST genes (*GSTA2, GSTA3, *and* GSTA4*) was detected in the liver of AFB1 + 10-HSA treated compared to AFB1-only animals (Fig. 2D). The resulting AFB1-glutathione conjugate can be eliminated as a waste product from the liver and does not form aflatoxin-DNA adducts (Fig. 2E) (46). Expression of these genes was significantly decreased in AFB1-treated animals compared to untreated controls (Fig. 2D). It is noteworthy that the *AKR7A5* gene was significantly upregulated in 10-HSA-treated animals (Fig. 2D). This gene in the mouse model is the functional ortholog of human gene aflatoxin B1 aldehyde reductase member 2 (*AKR7A2*), which is critical in detoxifying AFB1 in the liver (50). This protein is shown to work in conjunction with *EPHX1* (microsomal epoxide hydrolase, mEH) to promote the formation of AFB1-dialcohol, which can be eliminated from the liver as a waste product (Fig. 2E) (46). Gene *EPHX2* (soluble epoxide hydrolase, sEH) has been shown to promote AFB1 and NAFLD pathogenesis in the liver (7, 51). Although we observed increased levels of sEH transcripts in 10-HSA-treated animals, sEH protein quantity and activity in the liver were not changed (Fig. S3).

To further understand how 10-HSA treatment was activating the transcription of genes essential for AFB1 detoxification, we queried transcription factors regulating these genes. The TRRUST analysis showed significantly increased activity of PPARα and NRF2 in the liver with 10-HSA treatment (Fig. 1J). Excitingly, NRF2 regulates the expression of *AKR7A2,* alpha GSTs, and *EPHX1* (52–56). PPΑRα activation is known to promote NRF2 gene transcription, indicating that the activity of 10-HSA through promotion of PPARα led to an increase in NRF2 activity (57). We hypothesize that the activation of PPARα and NRF2 transcription factors in the liver of 10-HSA-treated animals leads to increased AFB1 detoxification and reduced the aflatoxin-DNA adduct formation, thereby reducing gene expression required for DNA damage response and repair (Fig. 2E).

### PPARα-mediated activation of liver regeneration gene expression

**Fig 3:**
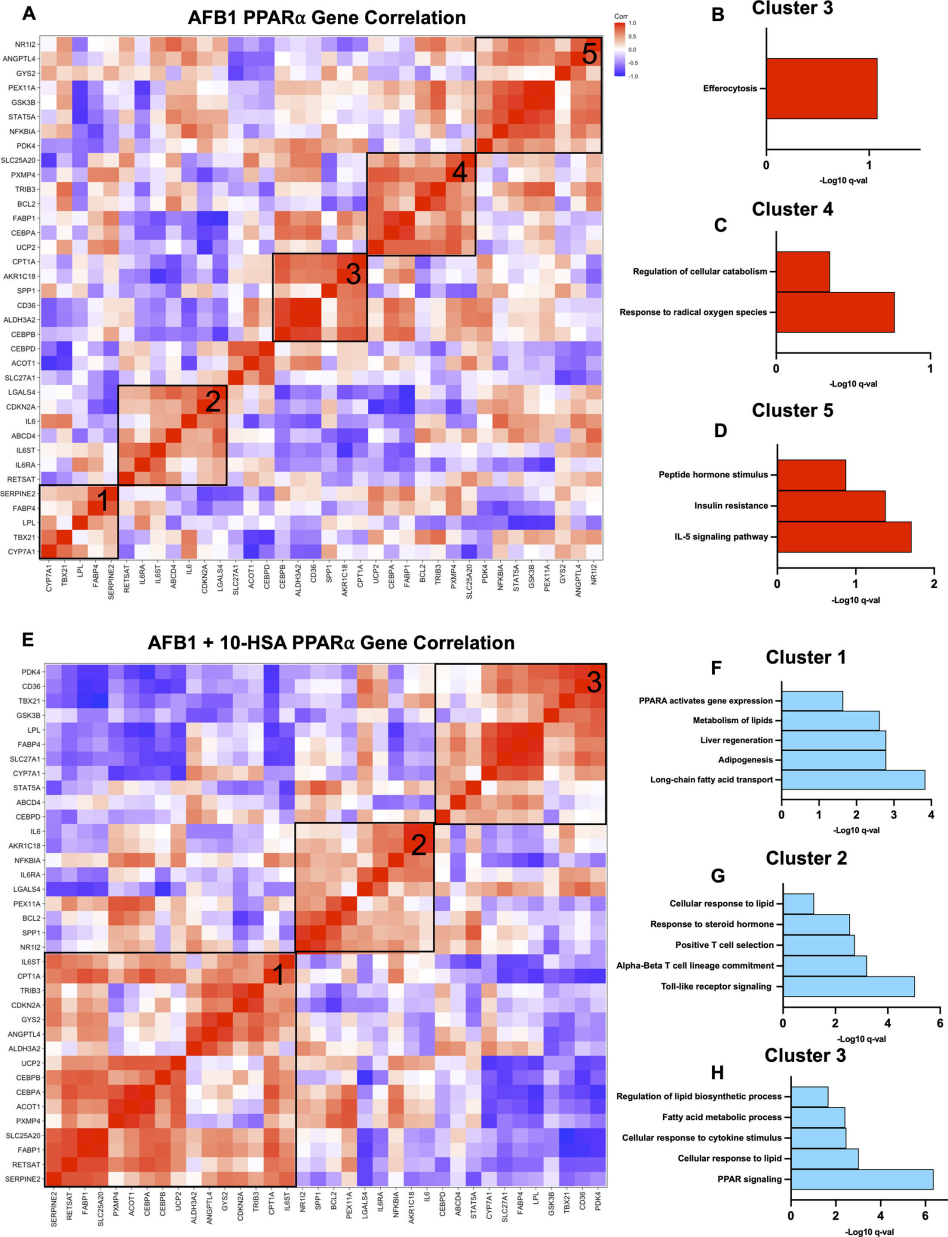
PPARα signaling promotes liver regeneration. (**A**) Pearson’s correlation plot of known PPARα-regulated genes based on the TRRUST database in AFB1-exposed mice from liver RNA-seq data. Pathway analysis of (**B**) Cluster 3, (**C**) Cluster 4, (**D**) and Cluster 5 from AFB1-exposed mice. (**E**) Pearson’s correlation plot of known PPARα-regulated genes AFB1 + 10-HSA mice from the liver. Pathway analysis of (**F**) Cluster 1, (**G**) Cluster 2, and (**H**) Cluster 3 from AFB1 + 10-HSA mice.

** **We sought to determine the molecular basis of the hepato-protective mechanisms of 10-HSA by analyzing the PPARα-induced gene expression patterns and associated functional networks in the liver of AFB1-exposed animals with and without 10-HSA treatment. Pearson’s correlation analysis revealed five major correlation clusters of PPARα-regulated genes in AFB1-exposed mouse livers (Fig. 3A). Cluster 1 pathway analysis showed PPAR signaling as the only pathway, and cluster 2 contained genes primarily involved in the regulation of MAPK1 activation (Fig. S4A and B). Efferocytosis was the only pathway to be detected in cluster 3 and could indicate the presence of a higher prevalence of apoptotic cells in the AFB1-exposed liver, which required clearance (Fig. 3B). Response to radical oxygen species was the primary pathway detected in correlation cluster 4 (Fig. 3C). AFB1 exposure and NAFLD are well recognized to drive oxidative stress, and our correlation analysis suggested that PPARα-based transcription was adapted in AFB1-exposed mice to promote genes that reduce oxidative stress (Fig. 3C). Cluster 5 showed a positive correlation of genes within IL-5 signaling and insulin resistance pathways (Fig. 3D). IL-5 signaling in the liver has been implicated in liver fibrosis, a common finding with AFB1 exposure effects (58, 59). Aflatoxin and NAFLD are known risk factors for the development of diabetes, and the positive correlation of genes responsible for insulin resistance may provide a mechanistic explanation (60).

Animals treated with 10-HSA showed distinct differences in PPARα-regulated gene correlation clustering patterns compared to AFB1-exposed only mice (Fig. 3E). There were three distinct clusters of positively correlated genes in 10-HSA-treated AFB1-exposed mice. Cluster 1 showed expected lipid metabolism, fatty acid transport, and adipogenesis pathways (Fig. 3F). Excitingly, genes associated with the liver regeneration pathway were also positively correlated (Fig. 3F). Cluster 2 also showed upregulation of the lipid response pathway (Fig. 3G). Additionally, cluster 2 highlighted immune cell pathways including TLR signaling and T cell selection and lineage commitment (Fig. 3G). While these pathways are not typically associated with liver function, it suggests that 10-HSA activated PPARα signaling may promote both innate and adaptive immune processes and overcome effects of the AFB1 exposure on immune dysfunction (7). Cluster 3 was primarily represented by positive correlation of genes mapping to lipid metabolism pathways (Fig. 3H).

In healthy control animals without 10-HSA treatment or AFB1 exposure, the pattern of PPARα-regulated gene clusters was similar to that detected in 10-HSA-treated animals (Fig. S5). Healthy control animals showed two distinct clusters of positive correlation (Fig. S5A). Liver regeneration, long-chain fatty acid transport, and oxidative stress-related genes all showed a positive correlation in cluster 1 (Fig. S5B). Cluster 2 showed a positive correlation of genes relating to lipid homeostasis, cholesterol metabolism, adipogenesis, and overall PPAR signaling (Fig. S5C). These findings demonstrate the similarities in PPARα-induced transcription by correlation analysis between 10-HSA-treated animals and healthy control mice, suggesting the recovery of AFB1-disrupted PPARα-regulated pathways by 10-HSA to normal levels. Positive correlation of liver regeneration and multiple pathways pertaining to lipid metabolism were not detected in the AFB1-treated mice correlation analysis.

Collectively, our data indicate a dramatic shift in PPARα signaling and associated gene expression in the liver of 10-HSA-treated mice compared to untreated AFB1-exposed mice. Animals administered 10-HSA during AFB1 exposure show increased correlation of lipid metabolism-related genes as well as liver regeneration and PPAR signaling and were comparable to healthy control mice. AFB1-exposed mice showed unique correlation of pro-inflammatory genes. Notably, the 10-HSA-treated mice did not show correlation of PPARα-regulated genes related to mitigating oxidative stress, while animals exposed to AFB1 without 10-HSA did. This indicates a reduced burden of oxidative stress due to 10-HSA treatment, leading to lack of a positive correlation with genes crucial for controlling oxidative stress. These data support the use of 10-HSA as a PPARα agonist to support homeostasis in liver fat metabolism and prevent NAFLD-like symptoms.

### Prevention of gut epithelial barrier damage and recovery of CD4+ T cell populations

**Fig 4:**
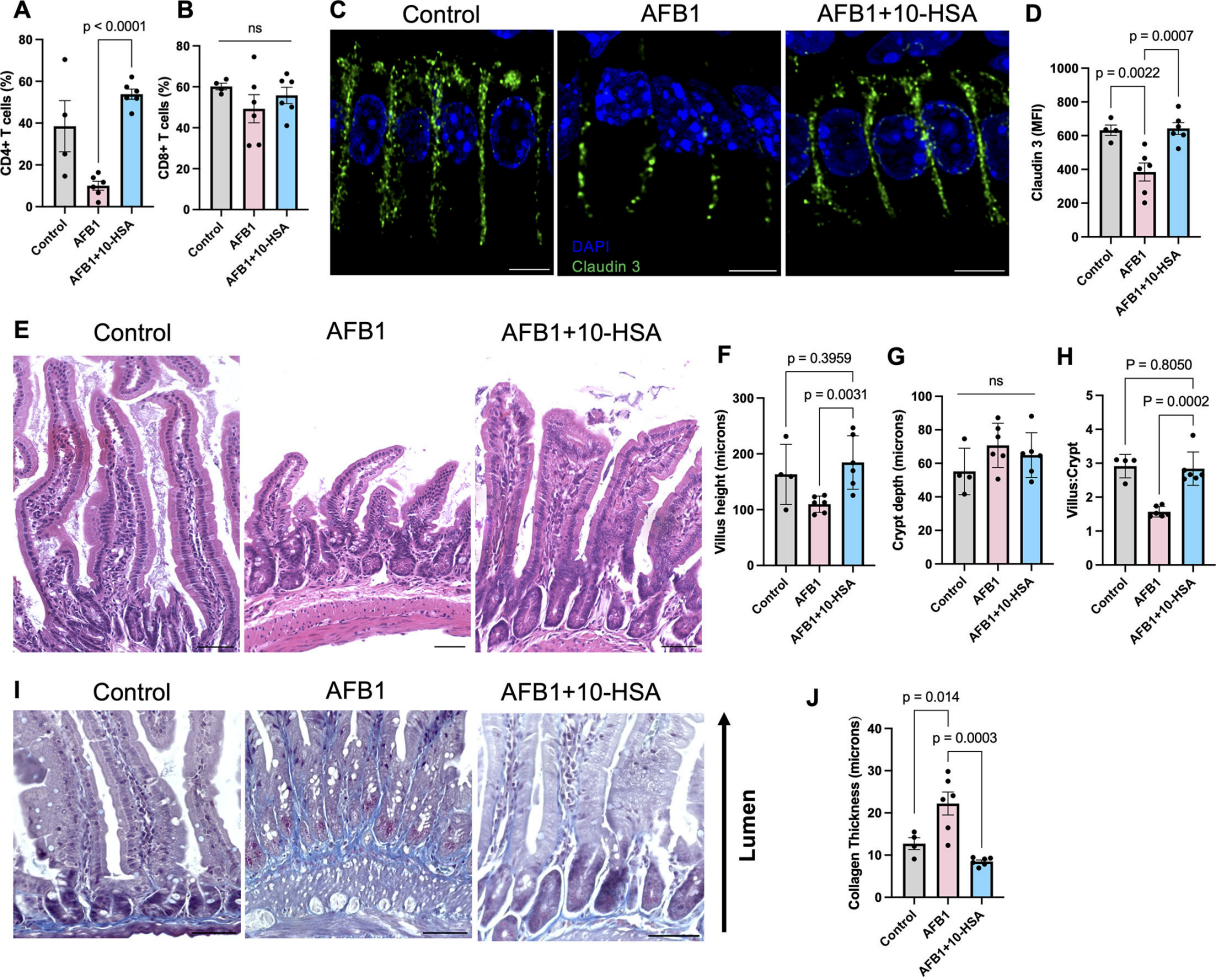
Prevention of gut epithelial disruption and fibrosis. Flow cytometry data showing (**A**) CD4+ T cell and (**B**) CD8+ T cell percentages from isolated LPLs. (**C**) Claudin-3 immunofluorescent stain representative images from the ileum. The scale bar represents 5 µm. (**D**) Semi-quantification of claudin-3 mean fluorescent intensity. (**E**) H&E stain of ileum representative images. Scale bar represents 50 µm. Quantification of (**F**) villus height, (**G**) crypt depth, and (**H**) villus:crypt ratio from H&E data. (**I**) Representative images of the trichrome-stained ileum. Scale bar represents 50 µm. (**J**) Quantification of collagen deposition by thickness in micrometers from trichrome staining.

AFB1 exposure causes reduced food intake and appetite due to alterations in gut morphology and function that drive weight loss (61). Increased weight gain with 10-HSA (Fig. 1C) supported stabilization of appetite potentially as a result of restored gut morphology and function. Thus, we sought to determine whether 10-HSA prevented disruption of gut epithelial barriers and mucosal cells during AFB1 exposure. Previous reports showed that AFB1 exposure reduces T-cell populations in the gut (7). We measured changes in the prevalence of gut mucosal CD4+ and CD8+ T cell subsets by flow cytometric analysis (Fig. S6). AFB1 exposure resulted in the loss of CD4 +T cell percentages compared to unexposed controls (Fig. 4A). The 10-HSA treatment significantly restored CD4+ T cell percentages compared to the AFB1-exposed group, and those were comparable to healthy unexposed controls (Fig. 4A). Changes in the CD8+ T cell populations were not so pronounced in AFB1-exposed versus unexposed groups (Fig. 4B).

NAFLD and AFB1 exposure have been shown to promote leaky gut by disrupting tight junction proteins of the epithelial barriers including claudin-3 (14, 62). To determine whether 10-HSA alleviated the impact of AFB1 on gut epithelial barriers, the ileum tissue was evaluated for tight junction protein claudin-3 expression and morphology by immunohistochemical analysis (Fig. 4C). Intact structural integrity of the epithelial barrier was detected in healthy control animals (Fig. 4C). In contrast, AFB1-exposed animals showed substantial epithelial barrier disruption, as demonstrated by decreased claudin-3 protein continuity between epithelial cells (Fig. 4C and D). The 10-HSA treatment resulted in epithelial barrier recovery compared to AFB1-exposed animals (Fig. 4C). Semi-quantification of fluorescent signals showed a significant increase in claudin-3 protein levels in AFB1 + 10-HSA compared to AFB1-exposed animals (Fig. 4D).

To understand the impact of AFB1 and 10-HSA on gut morphology, ileum sections were stained with hematoxylin and eosin (H&E) and analyzed (Fig. 4E). As expected, unexposed controls had long villus structures averaging above 150 µm in length (Fig. 4E and F). Animals exposed to AFB1 showed blunting of the villus structure (Fig. 4E and F). Animals receiving 10-HSA treatment during AFB1 exposure showed significant improvement in villus length (Fig. 4E and F). There was no detectable change in crypt depth between treatment groups (Fig. 4G). Due to the stunted villi in the AFB1-exposed group, the villus:crypt ratio was significantly decreased compared to unexposed healthy controls and animals receiving 10-HSA (Fig. 4H).

Potential thickening of the collagen layer was observed in the H&E-stained ileum tissue of AFB1-exposed mice (Fig. 4E). To determine whether 10-HSA reduced fibrotic changes in AFB1-exposed mice, Masson’s trichrome stain (MTS) assay was performed. Significantly thicker collagen deposition was detected in the sub-mucosal layer in AFB1 exposed mice compared to healthy controls (Fig. 4I and J). Treatment with 10-HSA showed a significant and remarkable decrease in collagen thickness in the sub-mucosa compared to AFB1-exposed-only animals (Fig. 4I and J). Collectively, these data suggest 10-HSA treatment protected against AFB1-induced gut epithelial barrier disruption and mucosal immunosuppression.

### Recovery of gut mucosal immunity and TGF-β and lipid metabolism gene expression

**Fig 5:**
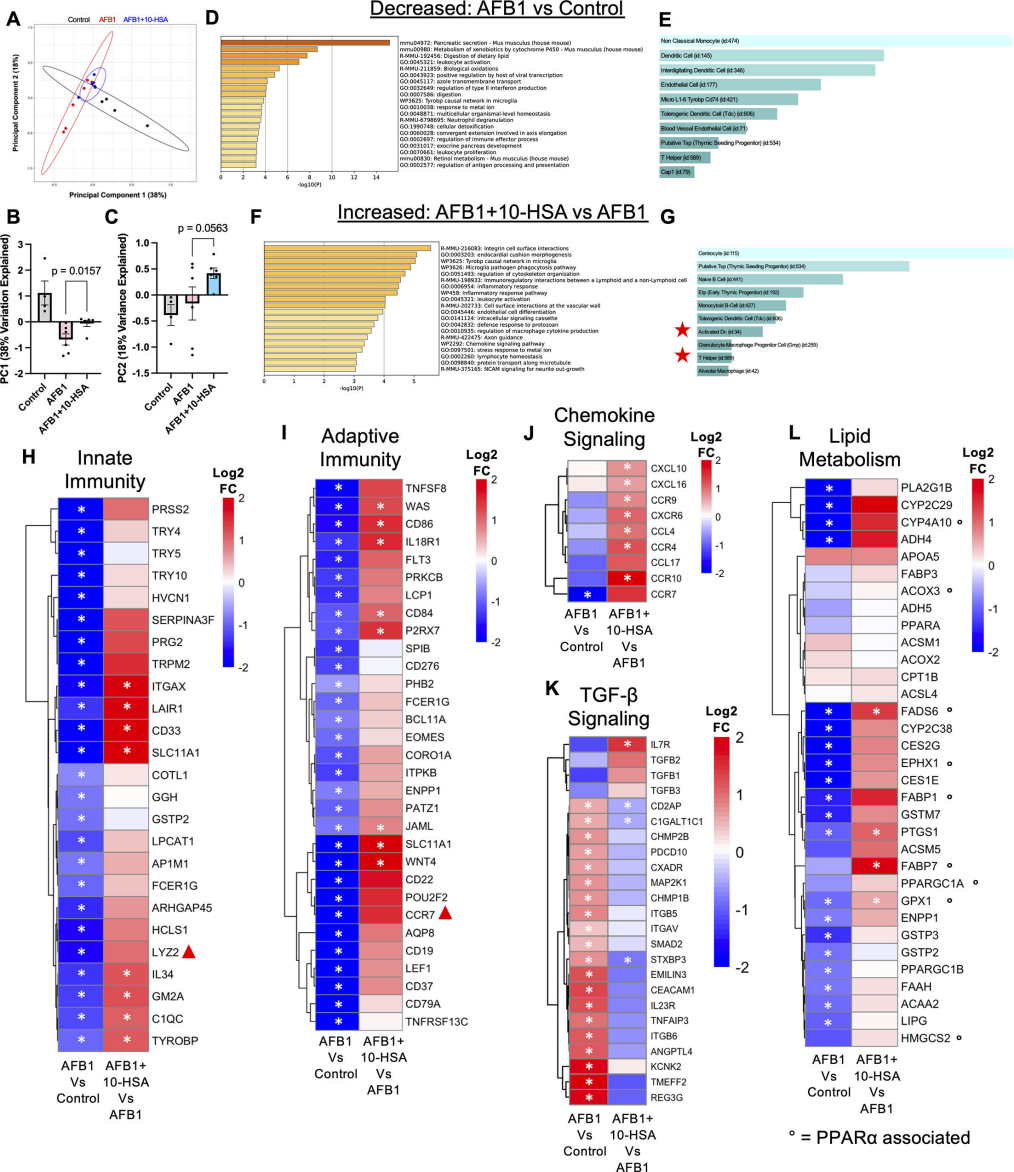
Activation of gut PPARα signaling and suppressed TGF-β production. (**A**) PCA plot of ileal RNA-seq data. Isolation of (**B**) PC1 and (**C**) PC2 to show separation between groups with statistical analysis. (**D**) Decreased expression pathways in AFB1-exposed mice compared to healthy control animals. (**E**) Decreased cell type signatures in AFB1-exposed mice compared to healthy control animals. (**F**) Increased expression pathways in AFB1 + 10-HSA mice compared to AFB1 animals. (**G**) Increased cell type signatures in AFB1 + 10-HSA mice compared to AFB1 animals. Heatmaps showing genes within (**H**) innate immunity, (**I**) adaptive immunity, (**J**) chemokine signaling, (**K**) TGF-β production, and (**L**) lipid metabolism pathways from gut RNA-seq data. Data in bar charts represent mean and standard error (*, *P* < 0.01).

 We sought to determine the 10-HSA treatment-driven molecular mechanisms underlying the gut tissue recovery. Transcriptomic profiles of ileal tissues by RNA-seq revealed tight clustering of 10-HSA-treated animals by principal component analysis (PCA), reflecting the treatment effect (Fig. 5A). AFB1 + 10-HSA-treated animals showed significantly altered localization along PC1 (38% data variation explained) compared to AFB1-exposed-only animals (Fig. 5B). PC2 also showed marked separation between AFB1 + 10-HSA and AFB1 animals (Fig. 5C). FDR correction of RNA-seq comparisons showed no significant changes between groups, so we utilized a *P* value ≤ 0.01 as a cutoff for pathway analysis. We first sought to understand the transcriptomic pathways suppressed by AFB1 exposure compared to unexposed healthy controls. The third-most enriched pathway downregulated by AFB1 was digestion of dietary lipids, indicating AFB1 exposure limits mucosal FAM (Fig. 5D). The type II interferon production pathways were also suppressed, indicating an immunosuppressive state driven by AFB1 exposure (Fig. 5D). Other immune-related pathways including neutrophil degranulation, immune effector process, and leukocyte proliferation also showed suppression with AFB1 exposure compared to unexposed controls (Fig. 5D). Cross-referencing the RNA-seq data with scRNAseq data sets revealed a decrease in a multitude of critical immune cell signatures due to AFB1 exposure (Fig. 5E). AFB1 exposure reduced cell signatures of naïve B cells as well as monocytoid B cells and T helper (CD4+) cells (Fig. 5E). These data confirm the immunosuppressive impact of AFB1, which is well documented in the literature and support our previous data showing reduced mucosal CD4+ T cell populations (7, 63). Critically, pathway analysis of genes upregulated by AFB1 compared to control animals revealed an increase in TGF-β production-related genes (Fig. S7A). TGF-β production and signaling drives fibrosis and immunosuppression (64, 65). These data support and explain our findings of increased collagen deposition in the gut of AFB1-exposed mice compared to control animals and those with 10-HSA treatment.

 Pathway analysis of genes with upregulated expression from AFB1 + 10-HSA treated compared to AFB1 animals revealed a remarkable impact on gut mucosal immunity. Immune cell interactions and overall inflammatory response potential in the gut were enhanced by 10-HSA treatment (Fig. 5F). Increased expression of the chemokine signaling pathway indicated that AFB1 + 10-HSA-treated animals could more effectively recruit immune cells to the gut to combat pathogens, as compared to AFB1-exposure-only animals (Fig. 5F). When cross-referencing genes with significant upregulation in response to 10-HSA treatment with scRNAseq data sets, an increase in monocytes and multiple dendritic cell subset signatures appeared, indicating the increased traffic and prevalence of immune cells into the gut (Fig. 5G). Increased expression of T helper (CD4+) cell transcriptional signature was noted during 10-HSA treatment, while it was downregulated in AFB1-exposed mice (Fig. 5E and G). These data indicate a striking shift in gut mucosal immunity of both innate and adaptive immune systems with 10-HSA treatment. These data also validate the findings about the significant increase in CD4+ T cell populations observed in the gut following 10-HSA treatment (Fig. 4A). A significant decrease in TGF-β production pathway in AFB1 + 10-HSA compared to AFB1 animals was also detected (Fig. S7B). These data further mechanistically explain our findings of decreased collagen deposition and increased immune cell activity in the gut of AFB1 + 10-HSA treated compared to AFB1 exposure only animals.

A detailed analysis was performed to identify genes contributing to the major changes in molecular pathways of gut immunity. In the innate immunity pathway, there was upregulation of the *LYZ2* gene (annotated with triangle, *P* = 0.027, log2FC = 0.97) in AFB1 + 10-HSA-treated animals, an innate anti-microbial peptide which was significantly decreased upon AFB1 exposure only (Fig. 5H). Lysozyme expression has been shown to reduce in the gut following AFB1 exposure (7). Expression of the *IL34* gene was significantly upregulated in AFB1 + 10-HSA compared to AFB1-exposed animals (Fig. 5H). IL-34 is critical in stimulating monocyte survival and is downregulated by AFB1, which contributes to decreased innate immune activity during AFB1 exposure (Fig. 5H) (66). There was also significant upregulation of *CD33* expression, a marker for myeloid cells, indicating an increased presence of this cell type in 10-HSA-treated mice (Fig. 5H).

In the adaptive immunity pathway, increased expression of *CCR7* (annotated with triangle, *P* = 0.025, log2FC = 1.49) in the gut of animals treated with 10-HSA (Fig. 5I) was detected. CCR7 is critical in stimulating Th17 cell differentiation, which promotes a functional and stable gut epithelium (67). A significant increase in *CD86* and *CD84* expressions in AFB1 + 10-HSA animals was detected, while these markers were significantly downmodulated in AFB1-exposed mice (Fig. 5I). They represent key markers for dendritic and hematopoietic progenitor cells, respectively (68, 69). Collectively, these data suggest the increased prevalence of dendritic cell populations capable of stimulating adaptive immune response through antigen presentation. Further analysis of the genes involved in the chemokine signaling pathway revealed a significant increase in *CXCL10/16, CCR4/9/10, CXCR6,* and *CCL4* gene expression in AFB1 + 10-HSA treated compared to AFB1-exposed-only animals (Fig. 5J). The increased expression of *CCR9* is of particular interest as it promotes mucosal T cell homing, while *CCR6* enables Th17 cell trafficking (70, 71).

In AFB1-exposed animals, expression of 20 genes related to TGF-β production was significantly upregulated compared to healthy control animals (Fig. 5K). AFB1 + 10-HSA-treated animals showed decreased levels of 17 of those 20 genes, three of which were significantly altered, compared to AFB1-exposed mice (Fig. 5K). The *IL7R* gene expression showed a significant increase in the AFB1 + 10-HSA treated compared to AFB1 exposure only (Fig. 5K). IL-7 signaling (mediated through IL-7R) is critical for maintenance of T cell survival and is downmodulated by TGF-β signaling, especially in memory CD4+ T cells (72, 73). However, as previously reported in the literature, *TGFB1* mRNA levels were decreased in the gut of AFB1-exposed mice (Fig. 5K) (7). This indicates the TBF-β production in the liver had a direct effect on the gut. Increased TGF-β signaling is also supported by data showing significantly increased collagen deposition in the gut of AFB1-treated mice, which was counteracted by 10-HSA treatment (23, 74). These data highlight the critical nature of the gut-liver axis, where TGF-β is produced in the liver and impacts signaling and morphology in the gut.

Due to the decrease in digestion and lipid metabolism pathways seen in AFB1-exposed mice, we examined whether 10-HSA treatment recovered those pathways (Fig. 5L). Genes critical for FAM *FABP7* and *FADS6* showed significantly increased expression with 10-HSA treatment (Fig. 5L). There was also a notable increase in *FABP1* (*P* = 0.13, log2FC = 1.57)*,* and *ACSM5* (*P* = 0.18, log2FC = 0.96) expressions in 10-HSA-treated animals compared to AFB1-exposed animals (Fig. 5L). There was an increase in nine known PPARα regulated and associated genes in AFB1 + 10-HSA compared to AFB1 animals, indicating an increase in PPARα signaling in the gut (Fig. 5L, annotated with circle). These data support our previous findings that LP, in part through the generation and secretion of 10-HSA, promotes PPARα activation (16). In addition, PPARα signaling and TGF-β signaling have been shown to inhibit each other (75, 76).

Evaluation of genes associated with epithelial structural integrity and function detected a significant increase in the expressions of *CLDN5*, *ICAM1,* and *ITGAX* in 10-HSA-treated animals compared to AFB1-exposed-only animals (Fig. S8). We also detected a significant increase in junctional adhesion molecule *JAM2* expression following 10-HSA treatment (Fig. S8). Increased expression of *MYD88*, a critical protein in the TLR receptor cascade, was detected in 10-HSA-treated animals compared to AFB1-exposed animals (Fig. S8).

Upon comparison of AFB1 + 10-HSA-treated animals with healthy control animals, upregulation of oxidative phosphorylation and digestion system process-related genes was noted (Fig. S9). These data demonstrate that even 10-HSA treatment with AFB1 exposure showed increased gene expression related to mitochondrial energy metabolism and gut function compared to healthy control mice. Together, our data show the dramatic impact of 10-HSA on gut mucosal immunity, energy homeostasis, and function of the gut during AFB1 exposure. These data also indicate that the AFB1-induced TGF-β production originating in the liver has a direct impact on gut homeostasis and morphology, again highlighting the critical role of the gut-liver axis.

### Activation of PPARα-regulated Th17- and IL-17-associated gene expression in the gut

**Fig 6:**
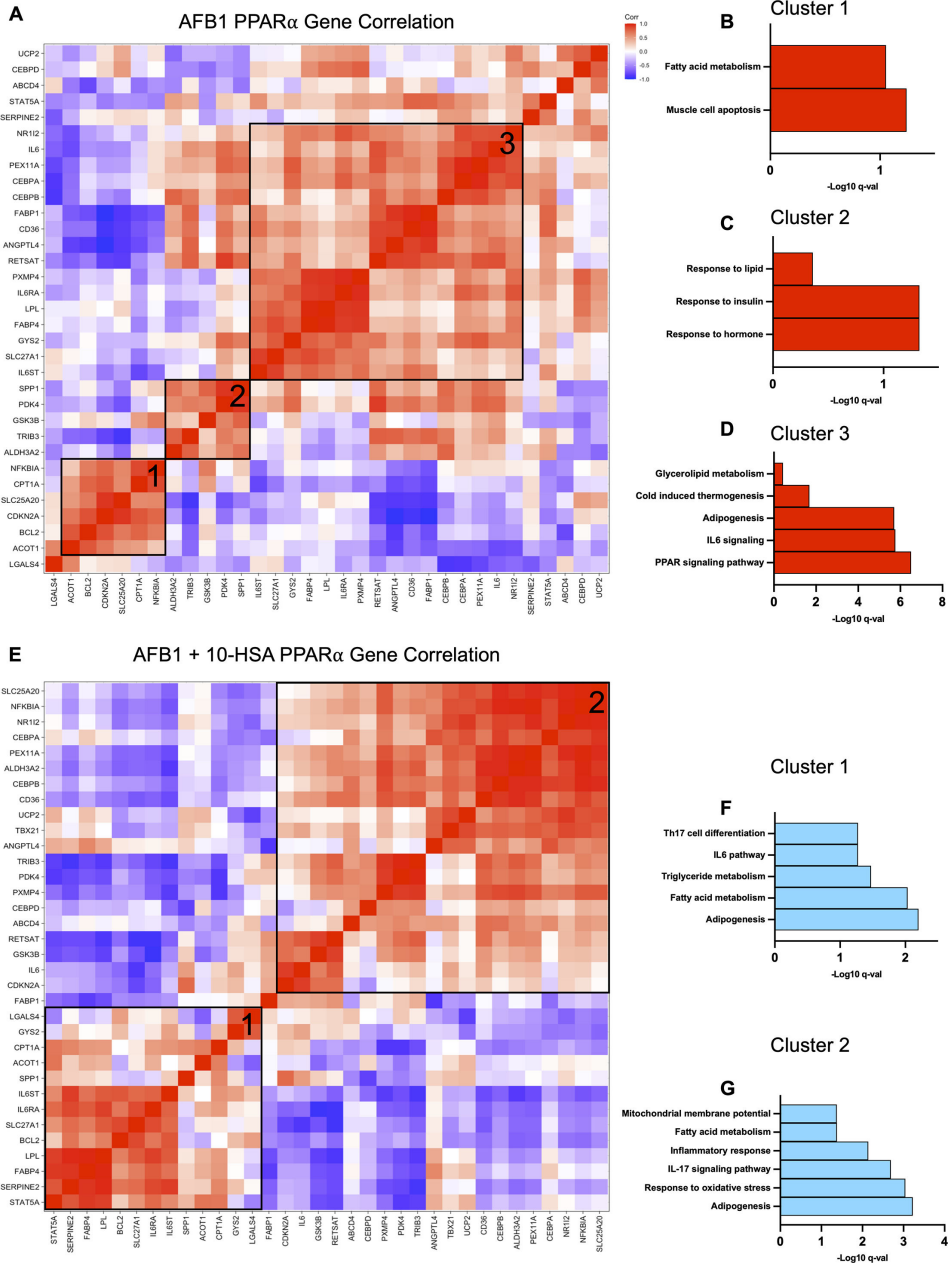
Activation of immunological gene expression patterns by PPARα. (**A**) Pearson’s correlation plot of known PPARα-regulated genes taken from the TRRUST database in AFB1-exposed mice from gut RNA-seq data. Pathway analysis of (**B**) Cluster 1, (**C**) Cluster 2, and (**D**) and Cluster 3 from AFB1-exposed mice. (**E**) Pearson’s correlation plot of known PPARα-regulated genes AFB1 + 10-HSA mice from gut RNA-seq data. Pathway analysis of (**F**) Cluster 1 and (**G**) Cluster 2 from AFB1 + 10-HSA mice.

** **To determine whether 10-HSA treatment modulated PPARα activation in the gut, we examined changes in PPARα-meditated gene expression and performed Pearson’s correlation analysis of RNA-seq data. Animals with AFB1 exposure showed three distinct clusters of positively correlated PPARα-regulated genes. Pathway analysis of cluster 1 showed correlation of FAM and muscle cell apoptosis genes (Fig. 6A and B). Genes *CPT1A* and *ACOT1* were positively correlated and are critical for FAM (Fig. 6A). In cluster 1, cytosolic inhibitor of NF-κB, *NFKBIA*, showed a positive correlation with *CPT1A* and *ACOT1* (Fig. 6A). Cluster 2 consisted of five genes corresponding to lipid response, insulin response, and hormone response pathways (Fig. 6C). Cluster 3 was the largest and consisted of genes regulating IL6 signaling, overall PPAR pathway, and adipogenesis (Fig. 6D).

** **Distinct differences in the correlation clustering pattern of PPARα-regulated genes were seen in AFB1 + 10-HSA-treated animals compared to animals without 10-HSA treatment, and this analysis identified two major clusters (Fig. 6E). Cluster 1 showed a positive correlation between genes regulating mitochondrial membrane potential, Th17 cell differentiation, IL6 signaling, and FAM-related pathways (Fig. 6F). Cluster 2 showed strong correlation of genes regulating response to oxidative stress as well as IL-17 signaling and FAM (Fig. 6F). AFB1 exposure is well documented to cause an increase in oxidative stress (77, 78). In 10-HSA-treated animals, PPARα-regulated genes associated with responding to oxidative stress (*ALDH3A2, CD36, CDKN2A, IL6,* and *UCP2*) showed a positive correlation (Fig. 6E and G). These correlative patterns were not seen in AFB1-exposed untreated mice, indicating 10-HSA was driving this shift in PPARα signaling and also drove the correlation of expressions of Th17- and IL-17-regulated genes, namely, *CEBPB, IL6, NFKBIA,* and *GSK3B* (Fig. 6E through G). IL-17 signaling is crucial for gut mucosal immunity as well as maintaining gut epithelial integrity (79). Similar correlation in gene expression was not observed in AFB1-exposed untreated mice (Fig. 6A).

Transcriptomic data from healthy control animals were interrogated for PPARα gene correlation patterns (Fig. S10). Control animals displayed two major clusters of positive correlation among known PPARα-regulated genes (Fig. S10A). Pathway analysis revealed cluster 1 showed a positive correlation of genes within adipogenesis and inflammatory response pathways as well as hemopoiesis (Fig. S10B). Cluster 2 showed a positive correlation of genes regulating tissue remodeling, response to oxidative stress, lymphocyte proliferation, and cellular lipid response (Fig. S10C). Healthy control animals and 10-HSA-treated mice shared more similarities in PPARα-activated transcription positive correlation clustering patterns compared to AFB1 animals. Additionally, 10-HSA-treated mice and control mice demonstrate increased inflammatory response and oxidative stress pathways regulated by PPARα, while AFB1-exposed mice do not show correlation patterns relating to these important pathways. Collectively, these data demonstrate a substantial shift in the transcriptional activity of PPARα with 10-HSA treatment, as demonstrated by vastly different correlative patterns of PPARα-regulated gene expression in 10-HSA-treated mice.

### Enhanced gut microbial diversity and production of a pivotal hepatoprotective metabolite

**Fig 7:**
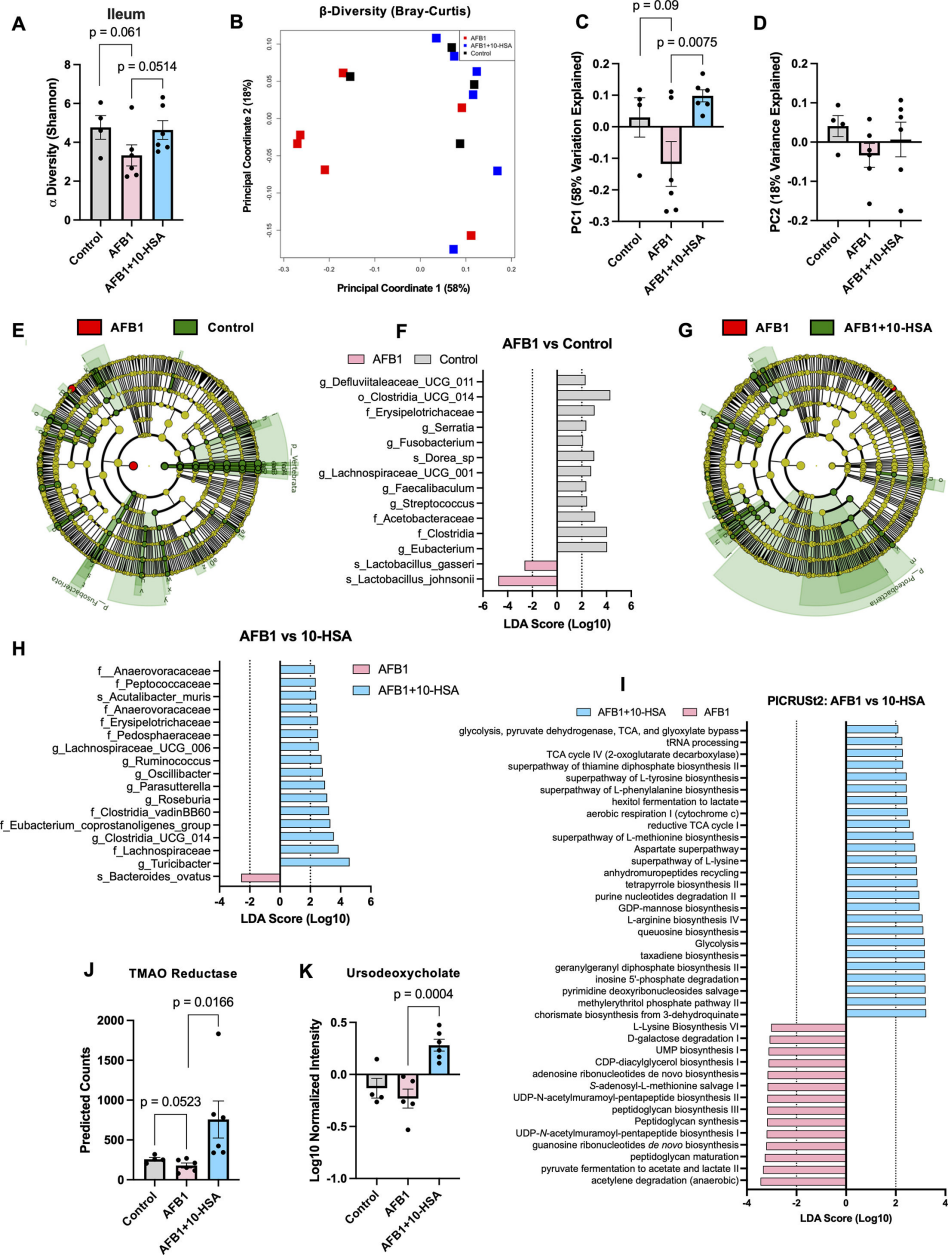
Restoration of gut microbiome diversity. (**A**) Shannon alpha diversity metric in the ileum of animals from each treatment group. (**B**) Beta-diversity, as measured by Bray-Curtis dissimilarity. Isolation of (**C**) PC1 and (**D**) PC2 to show separation between groups with statistical analysis from beta-diversity metrics. (**E**) LEfSe cladogram output comparing AFB1 and healthy control groups. (**F**) LEfSe LDA output comparing AFB1 and healthy control groups. (**G**) LEfSe cladogram output comparing AFB1 + 10-HSA and AFB1 groups. (**H**) LEfSe LDA output comparing AFB1 + 10-HSA vs AFB1 groups. (**I**) LEfSe LDA of PICRUSt2 data comparing AFB1 + 10-HSA vs AFB1 groups for predicted microbial enzyme pathways. (**J**) Predicted trimethylamine n-oxide counts in the three groups. (**K**) Ursodeoxycholate levels from metabolomic data compared between the three groups. Data in bar charts represent mean and standard error.

Aflatoxin exposure has been reported to disrupt gut microbiome composition and diversity (80). We monitored the composition of the gut microbiome at both the colon and ileum mucosal sites. Alpha diversity measurements indicated a reduction of diversity in the ileum of AFB1-exposed mice, while 10-HSA treatment restored microbiome diversity (Fig. 7A). Healthy control mice also showed increased alpha diversity in the ileum compared to those with AFB1 exposure (Fig. 7A). The colonic tissue showed no significant differences in microbial alpha diversity, while changes were evident in the small intestine (Fig. S11). Beta diversity measured by Bray-Curtis dissimilarity showed separation of AFB1-exposed mice from those treated with 10-HSA (Fig. 7B). Based on the data, healthy control mice clustered closer to 10-HSA-treated mice than to AFB1-exposed mice (Fig. 7B). Analysis of the principal coordinates (PC) from the Bray-Curtis dissimilarity plot showed significant separation of 10-HSA-treated animals from AFB1-exposed animals along PC1 (Fig. 7C). PC1 accounted for 58% of the variation seen in the data (Fig. 7C). PC2 showed no significant changes and only accounted for 18% of the variation seen in the data (Fig. 7D).

Linear discriminant analysis effect size (LEfSe) analysis of level 7 data from Qiime2 revealed significant (LDA > 2) changes in microbiome composition between AFB1-exposed mice and control mice (Fig. 7E and F). Cladogram analysis from LEfSe data showed enrichment of multiple nodes from control animals, while AFB1-exposed animals showed enrichment of only two, reflecting the reduction in microbiome diversity (Fig. 7E). LEfSe analysis revealed enrichment of *Streptococcus*, Lachnospiraceae, *Eubacterium*, and *Faecalibaculum* genera among others in the control-treated animals compared to those exposed to AFB1 (Fig. 7F). AFB1-exposed animals showed an increase in two *Lactobacillus* species compared to healthy control animals (Fig. 7F). *Lactobacillus* has been shown to remove aflatoxins from the gut, which may have granted these two species a competitive growth advantage (81). Overall, healthy control animals had higher gut microbiome diversity compared to AFB1-exposed mice.

We compared the ileal gut microbiome composition of AFB1-exposed mice to those treated with 10-HSA with LEfSe. The generated cladogram shows multiple nodes significantly enriched in the 10-HSA-treated compared to AFB1-exposed animals (Fig. 7G). Mice exposed to AFB1 without treatment showed enrichment of only one node compared to 10-HSA treated mice (Fig. 7G). LEfSe analysis of the microbial communities revealed *Turicibacter*, *Oscillibacter*, Lachnospiraceae, and *Roseburia* taxa as significantly enriched in 10-HSA-treated animals compared to AFB1 untreated mice (Fig. 7H). In AFB1-exposed mice, *Bacteroides ovatus* was the only taxon to show significant enrichment compared to 10-HSA-treated animals (Fig. 7H). Our findings suggest that AFB1-exposed animals have reduced gut microbial diversity, which was restored in 10-HSA treatment.

We next sought to understand the impact of gut microbiome changes on the microbial enzyme and metabolism pathways in the context of AFB1 and 10-HSA treatments. We utilized phylogenetic investigation of communities by reconstruction of unobserved states 2 (PICRUSt2) to predict bacterial enzyme counts in the gut microbiome based on the 16S sequencing. LEfSe analysis of the PICRUSt2 pathway output revealed a microbiome rich in TCA cycle activity and amino acid metabolism pathways for control animals compared to AFB1-exposed animals (Fig. S12). AFB1-exposed animals showed only two significantly enriched pathways pertaining to glucose and glycerol metabolism compared to healthy controls (Fig. S12).

Similar to healthy control animals, 10-HSA-treated animals showed a microbiome rich in TCA cycle activity and amino acid metabolism (Fig. 7I). Overall, 25 metabolic pathways were predicted to be significantly enriched in the 10-HSA-treated microbiome compared to animals exposed to AFB1 (Fig. 7I). Microbial fatty acid and glucose metabolism are crucial for synthesis of short-chain and other bioactive fatty acid molecules, which can have therapeutic effects on the gut epithelial lining. Animals exposed to AFB1 without 10-HSA showed increased predicted pathways pertaining to peptidoglycan synthesis and maturation compared to animals treated with 10-HSA (Fig. 7I). This indicates the microbial composition of AFB1-exposed animals is shifted to more gram-positive communities.

PICRUSt2 analysis revealed major changes in predicted enzyme enrichment in control animals compared to AFB1-exposed mice responsible for driving the changes in bacterial metabolism (Fig. S13A). We also observed major changes in predicted enzyme counts in 10-HSA-treated animals compared to AFB1-exposed mice (Fig. S13B). Importantly, we observed a significant increase in predicted counts of trimethylamine-N-oxide (TMAO) reductase in 10-HSA-treated mice, a bacterial enzyme capable of converting TMAO to trimethyl amine (TMA), thus detoxifying TMAO (Fig. 7J). Increased levels of TMAO are associated with cardiovascular disease (82). A strong trend of increased expression of this enzyme was seen in healthy control animals compared to AFB1 animals (Fig. 7J). Critically, *Lactobacillus* has not been shown to contain TMAO reductase (83). *Clostridium* and *Bacillus* taxa are both reported to contain TMAO reductase (83). Both healthy control and 10-HSA-treated animals show significant enrichment of members of those taxa compared to AFB1-exposed mice (Fig. 7F and H). These data suggest the reduction of TMAO in 10-HSA-treated animals is in part due to the preservation of microbiome diversity in the gut.

 We found a significant increase in ursodeoxycholate levels in the blood of 10-HSA-treated animals as compared to both healthy control and AFB1-exposed mice by utilizing untargeted metabolomics (Fig. 7K). This microbially derived metabolite is known to improve bile flow and improve cholestasis conditions (84). The pathway for its microbial synthesis is described in MetaCyc pathway PWY-7588. AFB1 is associated with cholestatic changes. It is likely that increased production of ursodeoxycholate during 10-HSA treatment may support microbiome diversity by maintaining effective bile acid flow and support gut functional recovery through restored microbiome. Collectively, our data suggest that 10-HSA treatment supports gut microbiome recovery and the restoration of homeostasis across the gut-microbiome-liver axis.

### Enhanced gut-liver functions evidenced by metabolomic profiling

**Fig 8:**
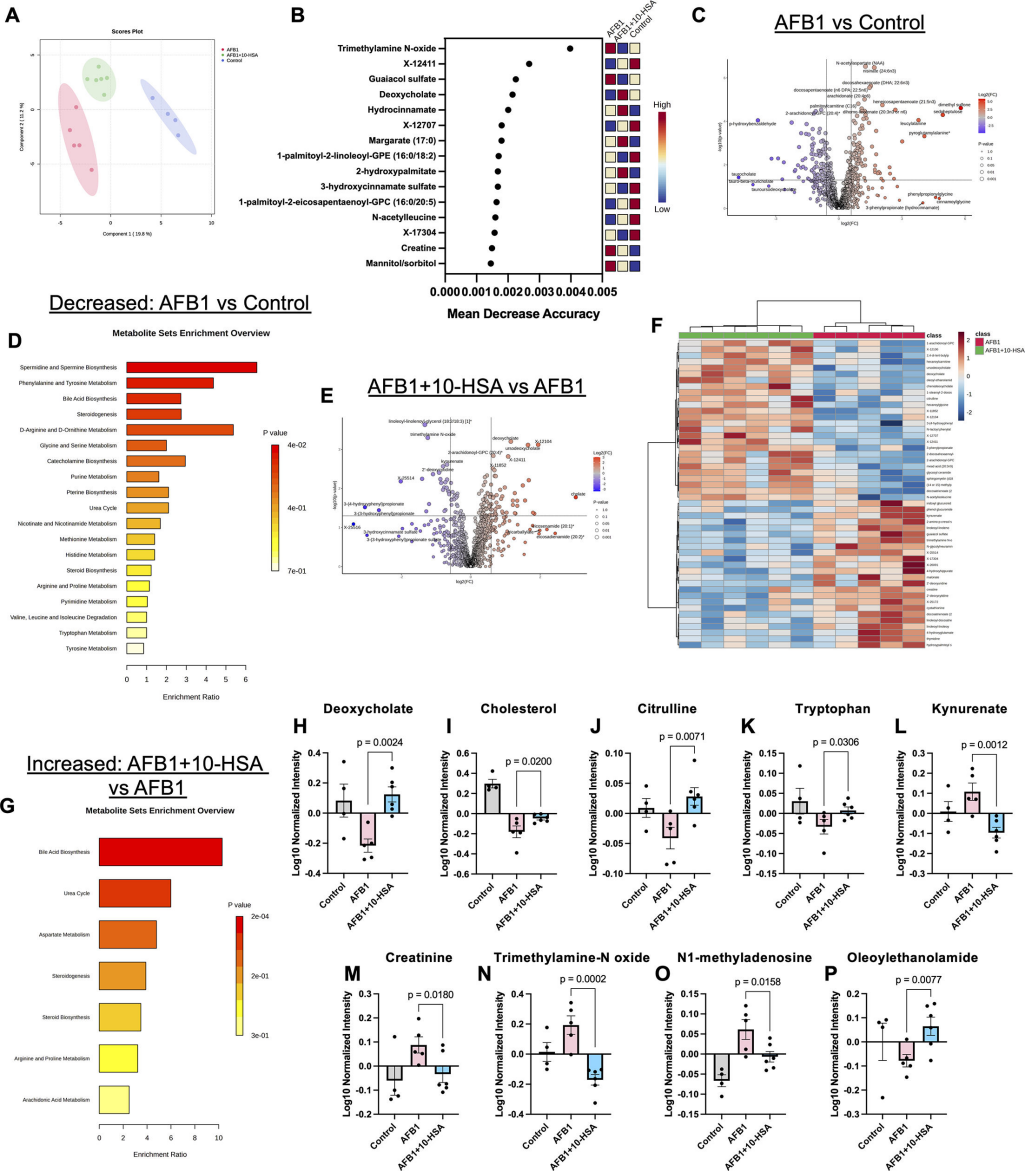
10-HSA treatment suppressed hepatocarcinogenic metabolite production and protected gut and kidney function. (**A**) PLS-DA plot of metabolomic data from AFB1, AFB1 + 10-HSA, and healthy control groups. (**B**) Random forest analysis of metabolomic data. (**C**) Volcano plot of significantly altered genes comparing AFB1 vs control metabolic profiles. (**D**) Pathways pertaining to decreased metabolites detected between AFB1 and control groups. (**E**) Volcano plot of significantly altered genes comparing AFB1 + 10-HSA vs AFB1 groups. (**F**) Heatmap of top 50 differentially abundant metabolites in AFB1 + 10-HSA vs AFB1 comparison. (**G**) Pathways pertaining to increased metabolites detected between AFB1 + 10-HSA and AFB1 groups. (**H–P**) Relevant metabolite levels indicating kidney, gut, and liver function across the three treatment groups.

** **Untargeted metabolomic profiling of peripheral blood samples was conducted to monitor the systemic impact of AFB1 exposure and 10-HSA treatment. PLS-DA showed distinct separation of treatment groups, especially along the component 1 axis (Fig. 8A). Random forest analysis of metabolite data sets from all three groups revealed TMAO as one of the most important metabolites for differentiating the metabolic signatures of each group (Fig. 8B). This metabolite was detected in high abundance in AFB1-exposed mice and in low abundance with 10-HSA treatment (Fig. 8B). This is supported by the gut microbiome data showing an increased TMAO reductase enzyme abundance within control and AFB1 + 10-HSA groups. In addition, hydrocinnamate, a gut microbiome-derived metabolite, showed a large impact on grouping accuracy (Fig. 8B). Hydrocinnamate is a key metabolite to indicate gut microbiome diversity, and it showed the highest levels in 10-HSA-treated animals (Fig. 8B).

A significant decrease (*P* < 0.05) in the levels of 140 metabolites was noted in AFB1-exposed mice compared to untreated controls (Fig. 8C). Pathway analysis of these metabolites demonstrated a significant decrease in bile acid biosynthesis (Fig. 8D). Blockage of the liver bile duct can lead to cholestasis, a commonly reported symptom of AFB1 exposure (10). In addition, decreased urea cycle and steroidogenesis-related metabolites were detected in AFB1-exposed animals (Fig. 8C). Aflatoxin has been reported to limit testicular steroidogenesis pathways, which has been associated with mitochondrial dysfunction (85). Multiple pathways relating to amino acid metabolism showed a marked decrease as well (Fig. 8D). Spermine and spermidine biosynthesis pathways were the most downregulated in AFB1-treated mice compared to control animals (Fig. 8D). Spermidine is known to activate transcription factor nuclear factor erythroid 2-related factor 2 (NRF2) to promote cellular oxidative stress response and mitochondrial biogenesis (86). NRF2 activity showed a significant decrease in the liver of AFB1-exposed mice compared to control animals (Fig. 1J).

 Comparing the metabolic profiles of 10-HSA-treated AFB1-exposed mice to AFB1-only-exposed mice without treatment revealed significantly (*P* < 0.05) increased production of 86 metabolites (Fig. 8E). Visualization of the top 50 altered metabolites between groups shows reversal of the AFB1-mediated depletion of key metabolites due to 10-HSA treatment (Fig. 8F). Overall, 10-HSA treatment increased bile acid presence in the blood, indicating its release from the liver (Fig. 8G). In addition, the steroidogenesis and urea cycle pathway showed increased metabolite levels in the 10-HSA-treated animals (Fig. 8G). These data indicate that 10-HSA was able to reverse the metabolic impact of AFB1 exposure and enhance gut-liver metabolism. Two metabolites critical in the bile acid synthesis pathway, deoxycholate and cholesterol, were significantly decreased in plasma samples of mice with AFB1 exposure (Fig. 8H and I). However, levels of these metabolites were significantly recovered by the 10-HSA treatment.

Citrulline is primarily synthesized in the mitochondria of gut enterocytes (87). We observed a significant reduction of citrulline in the peripheral blood of AFB1-exposed mice (Fig. 8J). This indicated impaired gut epithelium functionality through enterocyte mitochondrial dysfunction. Treatment with 10-HSA significantly restored citrulline levels (Fig. 8J). These results further support the data on the gut epithelial barrier recovery in AFB1-exposed mice with 10-HSA treatment.

Aflatoxin exposure has been reported to alter tryptophan metabolism (88). AFB1-exposed mice showed decreased levels of peripheral tryptophan levels, while 10-HSA treatment led to significantly elevated tryptophan levels (Fig. 8K). The levels of kynurenate, a downstream metabolite of tryptophan, were significantly elevated in AFB1-exposed mice compared to 10-HSA-treated animals (Fig. 8L). Increased tryptophan metabolism through the kynurenine pathway has been shown to drive Treg polarization and suppress immune activation (67). Reduced expression of the *IDO1* gene was seen in the 10-HSA-treated mouse gut (Fig. S14A). This gene codes for an enzyme critical for metabolizing tryptophan down the kynurenine pathway (67).

We utilized untargeted metabolomics data to evaluate kidney and liver functions. Creatinine, a metabolite typically removed from the body through kidney function, showed significantly elevated levels in AFB1-exposed mice (Fig. 8M). Kidney toxicity during aflatoxin exposure has been attributed mainly through increased oxidative stress (89). As expected, based on the random forest analysis, TMAO showed significantly elevated quantities in the AFB1-exposed mouse compared to those treated with 10-HSA (Fig. 8N). TMAO is synthesized in the liver from microbial TMA through conversion by flavin-containing monooxygenase 3 (*FMO3*) (90). Liver gene expression data showed a significant reduction in *FMO3* expression in 10-HSA-treated compared to AFB1-exposed animals (Fig. S14B). Calorie restriction has been shown to drive upregulation of *FMO3* in the liver (91). AFB1-exposed animals showed increased expression of *FMO3* compared to control animals (Fig. S14B). Therefore, *FMO3* upregulation in AFB1-exposed mice is potentially a result of the weight loss observed in this treatment group, which promoted increased circulating TMAO levels. N1-methyladenosine was also significantly upregulated in AFB1-exposed mice compared to those treated with 10-HSA (Fig. 8O) (92, 93).

Interestingly, a significant increase was noted in the production of oleoylethanolamide (OEA), a potent antioxidant, in 10-HSA-treated mice compared to those exposed to AFB1 only (Fig. 8P) (94). While the mechanism of the increase in OEA levels remains unclear, it is possible that fatty acid amide hydrolase activity responsible for the breakdown in OEA is reduced with 10-HSA treatment, leading to an increase in OEA accumulation. OEA has been touted for its ability to improve liver function and reduce hepatic oxidative stress and could be involved in the gut-liver axis recovery observed with 10-HSA treatment (95).

## DISCUSSION

** **The gut and liver are inextricably linked, and their crosstalk regulates key immunological and energy metabolism processes, which are disrupted by NAFLD. In the present study, we show that 10-HSA treatment preserved gut-liver axis functionality and promoted gut microbiome diversity in the AFB1 model for NAFLD. The adverse health effects of chronic AFB1 exposure and clinical disease are often not immediately apparent, leading to missed or delayed diagnosis or detection of AFB1 exposure in people. Prevention of AFB1 exposure-related damage through easily accessible interventions will be valuable for people living in areas known to be heavily contaminated with AFB1. Therefore, we developed the model of 10-HSA administration prior to AFB1 exposure to simulate the daily supplementation of this biotherapeutic in persons living in the areas of heavy AFB1 exposure risk. 10-HSA promoted epithelial barrier repair and function in the gut that coincided with increased PPARα signaling and lipid metabolism-related gene expression in the liver. Recovery of mucosal CD4+ T cells occurred along with upregulation of innate and adaptive immunity gene expression pathways in the gut of 10-HSA-treated mice. There was also a marked decrease in sub-mucosal collagen deposition due to 10-HSA treatment. Collagen deposition and fibrosis are regulated by TGF-β signaling in NAFLD (23, 64). We discovered that 10-HSA suppressed the significantly increased gut mucosal TGF-β signaling generated by AFB1 exposure. Simultaneously, we observed a significant increase in the expression of TGF-β in the liver of AFB1-exposed mice, indicating that the fibrosis in the gut was a result of TGF-β originating from the liver. Many agents that cause NAFLD or fibrotic liver disease promote hepatic TGF-β activity, indicating that this is a key therapeutic target to reduce liver disease pathology (96). Treatment with 10-HSA suppressed TGF-β production in the liver and pro-fibrotic hepatic stellate cell signatures, thereby reducing fibrotic changes and immunosuppression in the gut. We hypothesize that this reduction in TGF-β-mediated fibrotic changes in the gut and liver was mediated through increased PPARα signaling. It is known that PPARα and TGF-β exert a mutually inhibitory effect (20). In support of 10-HSA as a documented PPARα agonist, our data show increased hepatic and gut mucosal PPARα activation with 10-HSA treatment (28). These data demonstrate the remarkable recovery of the gut-liver axis by utilizing a PPARα activating therapeutic during NAFLD.

The liver is the predominant site of PPARα activity and is the primary target of AFB1-generated NAFLD damage, which drives steatosis, cholestasis, and carcinogenic changes (29, 30). We observed significantly reduced PPARα-regulated gene transcription in the liver of AFB1-exposed mice compared to control animals. This coincided with decreased expression of mitochondrial organization and oxidative phosphorylation pathways along with decreased FAM-related gene expression. NAFLD disrupts the structure and function of hepatic mitochondria, thereby driving pathogenesis (78, 97, 98). 10-HSA significantly revived lipid metabolism signaling in the liver and promoted genes related to mitochondrial ETC and organization. This indicates 10-HSA reinforces critical pathways which, when disrupted, drive NAFLD. Transcription factors PPARα and NRF2 showed significantly increased activity due to 10-HSA treatment. Activation of these transcription factors has been shown to limit liver injury and safeguard hepatic mitochondria by reducing fibrotic changes and limiting oxidative stress accumulation (99).

 The liver in AFB1-exposed animals showed marked upregulation in DNA damage response, double-strand break repair, and nucleotide excision-related gene transcription. After conversion of AFB1 to the 8,9-epoxide form by cytochrome p450, aflatoxin-DNA adducts form, which drive the formation of hepatic tumors (46). These adducts lead to DNA damage and double-stranded breaks, requiring repair (100). The cellular response includes nucleotide excision to remove aflatoxin-DNA adducts and the conduction of double-stranded break repair. Animals exposed to AFB1 showed significant upregulation of these DNA repair pathways. AFB1 + 10-HSA treatment showed a significant decrease in DNA repair pathways, indicating a decreased formation of aflatoxin-DNA adducts requiring excision and repair. Simultaneously, 10-HSA treatment promoted the transcription of genes critical in detoxifying AFB1 from its 8,9-epoxide form into excretable metabolites like AFB1-dialcohol. Dialcohol and glutathione metabolites of AFB1 8,9-epoxide do not form DNA adducts and thus reduce the requirement for cellular machinery to respond to this damage (46). The transcription of these AFB1 detoxifying genes (*GSTA2-4, EPHX1, ARK7A5*) is regulated by transcription factor NRF2, which was significantly upregulated in its activity in the liver during 10-HSA treatment. Activators of PPARα stimulate NRF2 activity (57). Given the significant increase in fatty acid oxidation pathways as well as known PPARα-regulated gene transcription during 10-HSA treatment, we hypothesize that the AFB1 detoxifying capabilities in the liver ultimately stem from the significant PPARα activation by 10-HSA treatment.

We evaluated the metabolomic profile to monitor production of key metabolites associated with gut function and liver metabolism. Low levels of peripheral deoxycholate, a bile acid, are associated with diarrhea and cirrhosis, known symptoms of AFB1 exposure (101, 102). Deoxycholate was significantly upregulated with 10-HSA treatment compared to untreated animals. These levels were comparable to control animals, indicating reversal to physiologically normal levels of bile acid-related metabolites due to 10-HSA treatment. Significant upregulation in citrulline levels was observed due to 10-HSA treatment. Citrulline is synthesized almost exclusively by gut enterocytes, and increased levels of this metabolite are reflective of restored gut function (103). Trimethylamine N-oxide (TMAO) was significantly reduced in 10-HSA-treated animals compared to AFB1-exposed animals. TMAO has been shown to be significantly upregulated in NAFLD conditions due to altered gut microbial composition and activity (104, 105). Increased TMAO can lead to cardiovascular disorders.

NAFLD decreases gut microbiome diversity in part through increasing gut fibrosis (106). Expectedly, AFB1-induced NAFLD decreased gut microbial diversity (107). The 10-HSA treatment promoted microbiome diversity as well as taxa containing the TMAO reductase enzyme. Our data indicate that the maintenance of a diverse microbiome is critical to decreasing TMAO levels during liver disease. During 10-HSA treatment, gut microbiome diversity was preserved, which increased the levels of the TMAO reductase enzyme present. This might have increased the microbiome functionality for clearing TMAO from the organism and reducing TMAO levels in peripheral blood. There was decreased expression of TMAO-producing enzyme *FMO3* in the liver following 10-HSA treatment. Our study demonstrates the highly interconnected gut microbiome-gut-liver axis can be therapeutically leveraged by a microbial metabolite to promote homeostatic recovery in multiple compartments. Furthermore, microbial metabolite ursodeoxycholate was elevated with 10-HSA treatment. This metabolite plays a key role in preventing cholestasis, a known symptom of AFB1 (10, 84, 108). The reduced cholestasis was evidenced by increased bile acid metabolites in the blood of 10-HSA-treated mice. Collectively, the maintenance of circulating levels of citrulline as well as the protection of gut microbial diversity due to 10-HSA treatment indicates profound recovery of the gut mucosal compartment.

In summary, our findings position 10-HSA as a strong microbial biotherapeutic candidate to preserve homeostasis across the gut-liver axis through activation of PPARα and FAM repair in multiple NAFLD. This therapeutic approach demonstrates the innovative use of a microbially derived metabolite to promote tissue repair pathways and restore lipid metabolism independent of live probiotics and enables us to pursue novel strategies to cure liver disease.

## MATERIALS AND METHODS

### Study design

 C57BL/6 male mice (8 weeks old) were purchased from Charles River and were maintained in a standard animal facility. After receipt, the mice were acclimated in the vivarium for 3 days prior to the study. During the study, all mice were maintained with a standard chow diet *ad libitum*. Mice were randomly assigned into three groups. One group (*n* = 6) was pretreated with 10-HSA (AstaTech A10837) at 100 mg/kg/day in vehicle for one week and then aflatoxin-β1 (Sigma-Aldrich A6636) dissolved in DMSO (final concentration in water was 0.1%) was added to their drinking water for 21 days at a concentration of 5 mg/L. Another group (*n* = 6) was pretreated with the vehicle control for 1 week and then aflatoxin-β1 was added to their drinking water for 21 days at a concentration of 5 mg/L. The third group (*n* = 4) served as the negative control group and received vehicle for 1 week prior to DMSO addition to drinking water for 21 days. After 21 days, mice were sacrificed in accordance with IACUC protocols, and the tissue was isolated and immediately stored as required for further analysis. We selected 100 mg/kg/day of 10-HSA after review of literature, which articulates known production of 10-HSA in physiologic conditions in addition to reviewing literature pertaining to clinical trials utilizing other fatty acids as oral therapeutic interventions to gut-associated disorders (109, 110). This dosage provides a starting point to facilitate the understanding of how this microbially derived biotherapeutic may be used to target the gut-liver axis in NAFLD.

The rhesus macaque study from Fig. 1A is as previously published (16).

### Immunofluorescence

Mouse ileum was isolated at necropsy and immediately submerged in fresh 4% paraformaldehyde (PFA) for 24 hours at room temperature, at which point it was moved to a 70% ethanol solution. The ileum was embedded in paraffin wax and cut at 5 µm. Slides were baked in a 60°C oven for 60 minutes and then deparaffinized with xylene and decreasing concentrations of ethanol in water. Slides were permeabilized with 1% Triton in 1× PBS for 25 minutes, and antigen retrieval was performed using a vegetable steamer (Oster 5711) with IHC Antigen Retrieval Solution high pH (pH 9; Invitrogen 00-4956-58). Slides were blocked with 10% goat serum in 1× PBS overnight, then claudin-3 primary antibody (ThermoFisher 34-1700) was incubated at room temperature at 1:200 dilution in 10% goat serum blocking buffer. Slides were washed and stained with goat anti-rabbit AF488 secondary antibody (Invitrogen A-11034) at 1:400 concentration in blocking buffer. Slides were incubated with DAPI to stain nuclei at 1:25,000 dilution and mounted with ProLong Diamond Antifade Mountant (ThermoFisher P36965) and allowed to dry at room temperature overnight.

### Hematoxylin and eosin staining

Mouse ileum was isolated as described above and cut at 5 µm thickness. Slides were deparaffinized, as described above. All steps mentioned included a wash step with DI water between steps. Fisherfinest Hematoxylin 1 was applied for 2 minutes, Clarifier (Epredia 7401) was applied for 20 seconds, Bluing (Epredia 7301) was applied for 1 minute, eosin-Y (Epredia 7111) was applied for 1 minute, and slides were dehydrated in 100% ethanol and cleared with xylene for 1 minute. Slides were then mounted with Permount (Fisher Chemical SP15-100) and imaged.

### Trichrome staining

Sections were treated in 60°C Bouin’s solution for 1 hour, followed by Wiegerts hematoxylin for 5 minutes, Beirich Scarlet/acid fuchsin solution for 15 minutes, phosphomolybdic/phosphotungstic acid solution for 15 minutes, aniline blue solution for 10 minutes and acetic acid (1%) for 5 minutes and then dehydrated with ethanol and cleared with xylene. The method was conducted using a tissue stain kit (Abcam AB150686). Slides were mounted and allowed to dry before imaging.

### Soluble epoxide hydrolase activity

Liver tissue (0.01–0.3 g) was suspended in chilled 2 mL sodium phosphate buffer (20 mM, pH 7) containing 5 mM EDTA, 1 mM PMSF, and 1 mM DTT. The tissue was homogenized with a Polytron (15 seconds twice at setting 6 with 15 seconds rest on ice between). Homogenates were centrifuged at 1,000 × *g* for 20 minutes at 4°C. Supernatants were transferred into a clean microcentrifuge tube and stored at −80°C. sEH activity was measured using t-DPO as the substrate at 37°C (111). Extracts were diluted 500-fold in NaPO4 buffer (0.1 M, pH 7.4) containing 0.1 mg/mL BSA. The diluted solution was incubated with the radioactive substrate ([S]final = 50 µM) for 30 minutes at 37°C. The amount of diols was quantified as previously described (111). sEH protein quantity was conducted with ELISA. Extracts were diluted 1,000-fold in 1× PBS and amount of sEH protein quantified as previously described (112).

### Flow cytometry

Lamina propria lymphocytes were isolated from the mouse ileum tissue through two 45 minute incubations in collagenase (Sigma Aldrich C6885 1 mg/mL in RPMI) medium at 37°C with agitation. The solution was strained with a 70 µm cell strainer and centrifuged for 5 minutes at 1,800 RPM. Cells were resuspended in 10% DMSO, 90% FBS freezing media, and frozen at −80°C and moved to liquid nitrogen. For flow cytometry staining, cells were thawed and rested at 37°C. Cells were washed with cold 1× PBS and then stained with Live/Dead Aqua (Thermo Scientific L34957), anti-CD45-AF488 (Thermo Scientific 53-0451-82), anti-CD3-AF700 (BioLegend 100215), anti-CD4-BV650 (BioLegend 100545), and anti-CD8a-PE/Dazzle-594 (BioLegend 100761) pre-conjugated flow antibodies. Cells were washed and then fixed in 2% PFA overnight. Cells were run through a flow cytometer (BD FACSymphony A3) at the California National Primate Research Center, and data were collected. FlowJo (v10.10.0) was used to gate.

### Microscopy

H&E and trichrome staining was imaged on the brightfield mode of the EVOSFL Auto 2 at 20× magnification (Thermo Fisher). Claudin-3 immunofluorescence was imaged with the Leica TCS SP8 STED 3X confocal microscope at 100× magnification at the Advanced Imaging Core at UC Davis. Images were deconvoluted with Huygens Deconvolution. Raw image files were used for semi-quantitative mean fluorescence intensity analysis in ImageJ (v2.14.0/1.54 f).

### RNA-seq and gene expression analysis

Ileum was preserved in RNAlater at −20°C after necropsy. The liver was preserved at −80°C after necropsy. RNA extraction for sequencing was performed as previously described (113). Sequencing was performed on AVITI sequencer at the UC Davis DNA Tech Core. Approximately 4 million reads per sample were collected with a Q30 > 95%. Sequences were aligned to the mouse genome (mm10) using STAR alignment, and reads per gene were output. Reads per gene files were organized and run through the limma.voom differential expression analysis pipeline in R. Differentially expressed genes were input into Metascape.org for pathway analysis. Pearson’s correlation analysis utilized known PPARα-regulated genes from the TRRUST database. Pearson’s correlation was conducted in R.

### Metabolomics and analysis

Untargeted metabolomic profiling of peripheral blood plasma was performed by Metabolon Inc. Plasma (100 to 250 μL) was collected per animal and submitted. One animal from the AFB1-exposed group did not give enough plasma for analysis; therefore, only five samples were submitted. Data were normalized and analyzed as previously described utilizing the MetaboAnalyst platform (16). Data were generated based on significant (*P* < 0.05) changes in metabolomic profiles between groups.

### 16S seq and analysis

Colon and ileum samples preserved in RNAlater at −20°C after necropsy were manually digested, and extraction was conducted as previously described (114). QIIME2 analysis was utilized to determine Bray-Curtis beta diversity and Shannon alpha diversity data. LEfSe analysis of level 7 QIIME2 taxonomic data was used to generate cladogram and LDA plots based on LDA > 2 cutoff for significance. PICRUSt2 was utilized from raw Fastq files to predict enzyme abundance in the microbiome.

### Statistical analysis

Data shown represent the mean ± SEM calculated by using all data points unless otherwise stated. Statistical significance was determined through unpaired *t*-tests when comparing values between two groups. One-way ANOVA was used to determine treatment effects between multiple groups. *P*-values of <0.05 were considered significant. All analyses were performed on GraphPad Prism (v9.4.0).

## Data Availability

Sequencing data generated for this study are available in Sequence Read Archive (SRA) under accession number PRJNA1249523.

## SUPPLEMENTAL MATERIAL

Supplemental Material mbio.01718-25-s0001.pdf Fig. S1 to S14.

ASM does not own the copyrights to Supplemental Material that may be linked to, or accessed through, an article. The authors have granted ASM a non-exclusive, world-wide license to publish the Supplemental Material files. Please contact the corresponding author directly for reuse.
