# Supplementary material for: Microbial biotherapeutic metabolite alleviates liver injury by restoring hepatic lipid metabolism through PPARα across the gut-liver axis
Source: mBio. 2025 Aug 12;16(9):e01718-25. doi: 10.1128/mbio.01718-25 (PMC12421843; doi:10.1128/mbio.01718-25)
Supplement: Supplemental Material — Fig. S1 to S14. [file mbio.01718-25-s0001.pdf]

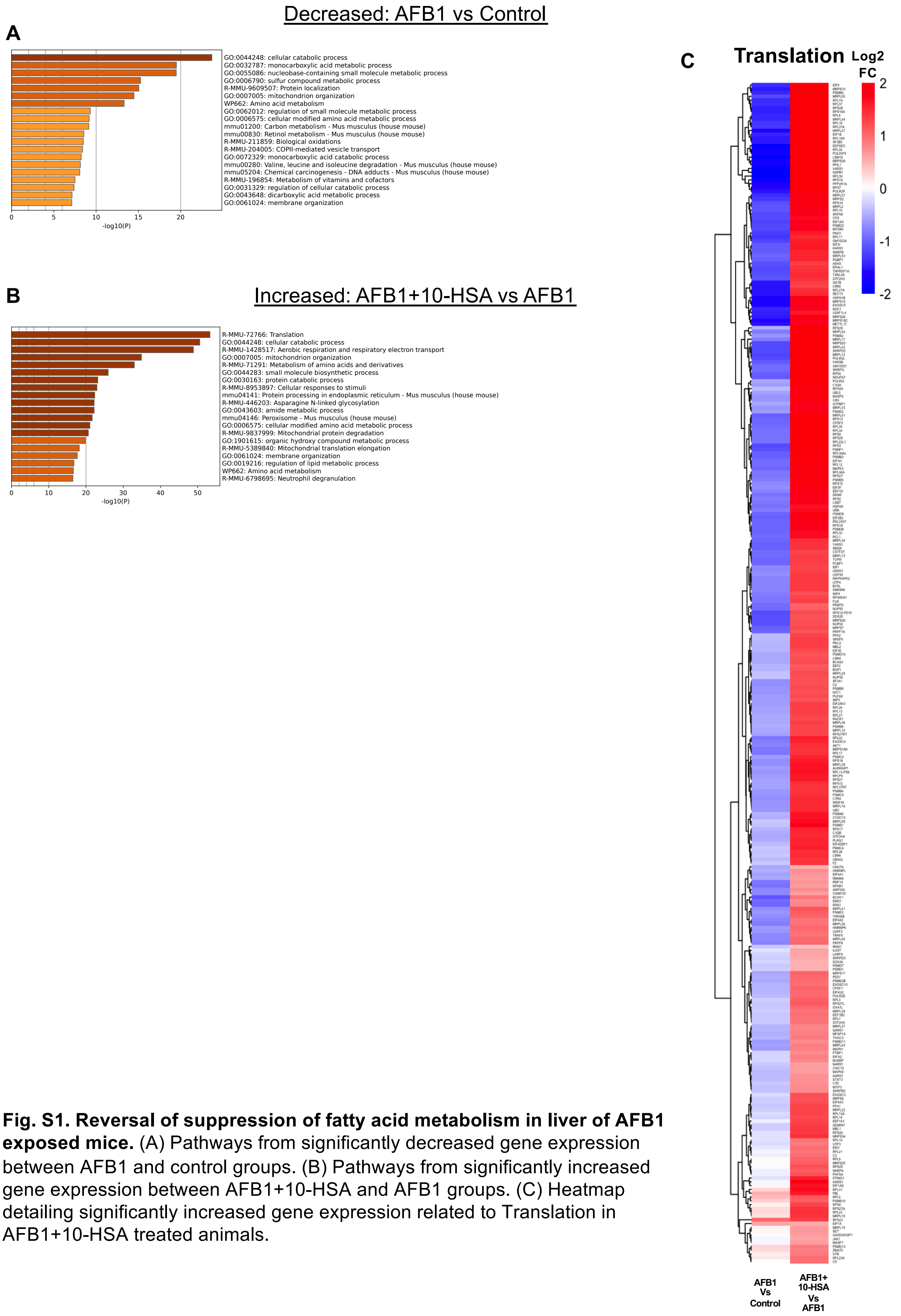

Fig. S1. Reversal of suppression of fatty acid metabolism in liver of AFB1 exposed mice. (A) Pathways from significantly decreased gene expression between AFB1 and control groups. (B) Pathways from significantly increased gene expression between AFB1+10-HSA and AFB1 groups. (C) Heatmap detailing significantly increased gene expression related to Translation in AFB1+10-HSA treated animals.

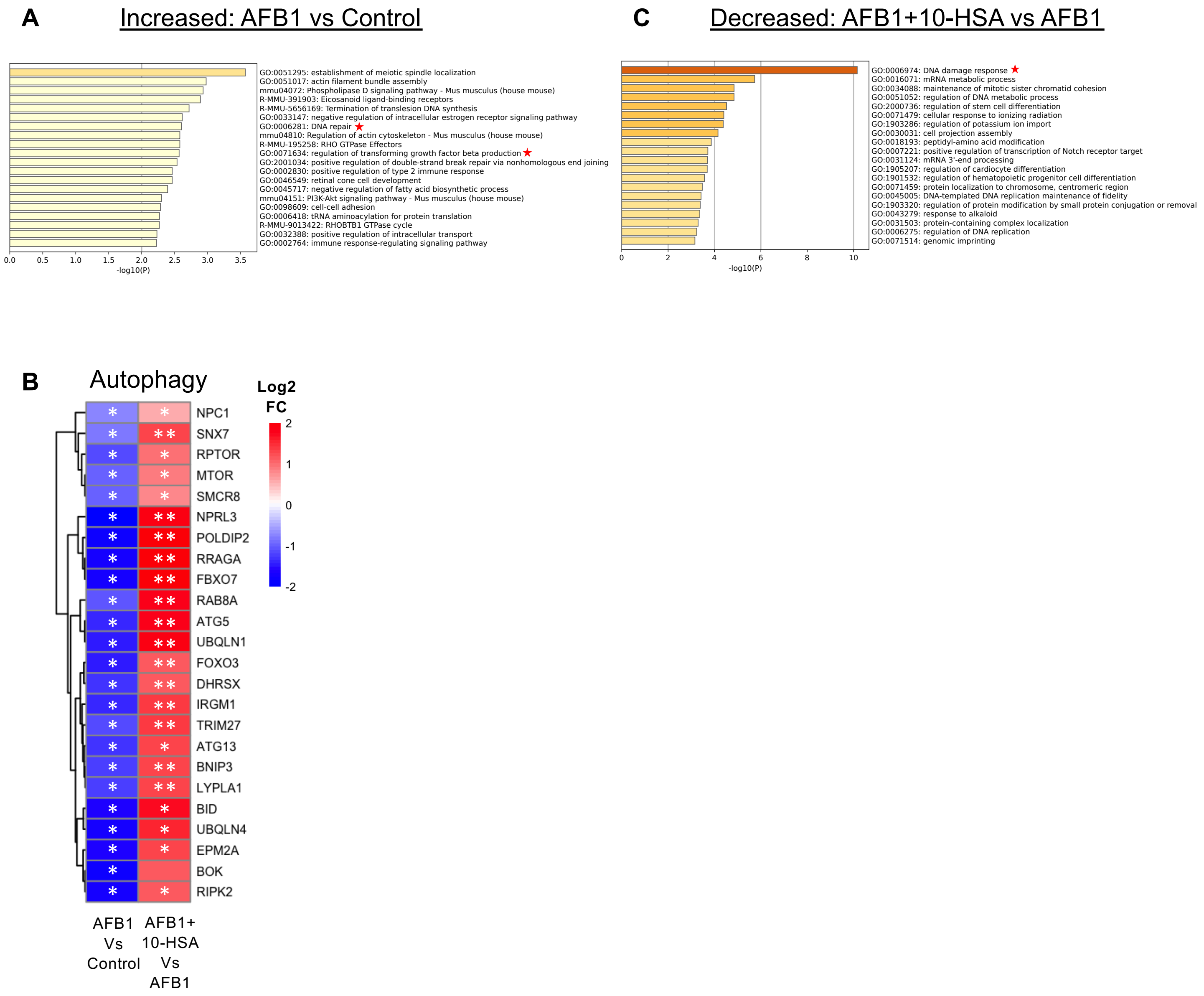

Fig. S2. Reduction of hepatic DNA repair requirements in AFB1 exposed mouse livers. (A) Pathways from significantly increased gene expression between AFB1 and control animals. (B) Autophagy related gene expression (C) Pathways from significantly decreased gene expression between AFB1+10-HSA and AFB1 groups.

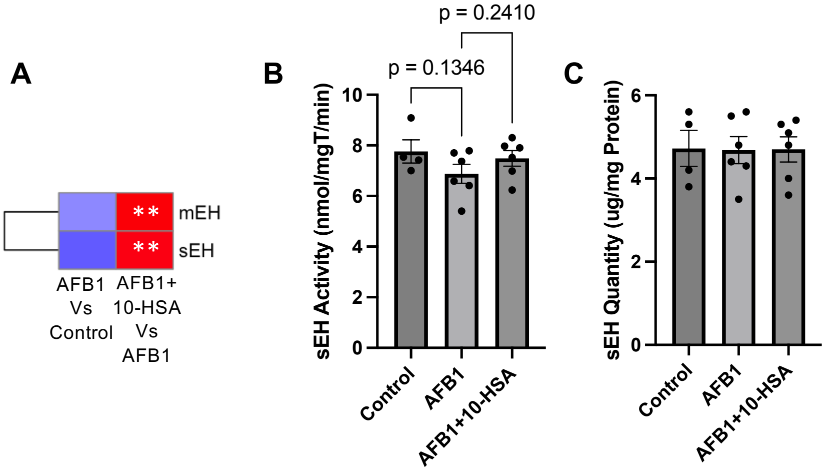

Fig. S3. 10-HSA did not increase sEH activity in liver of AFB1 exposed mice. (A) RNAseq data showing sEH and mEH levels between groups. (B) sEH activity. (C) sEH protein quantity. (** = FDR < 0.01).

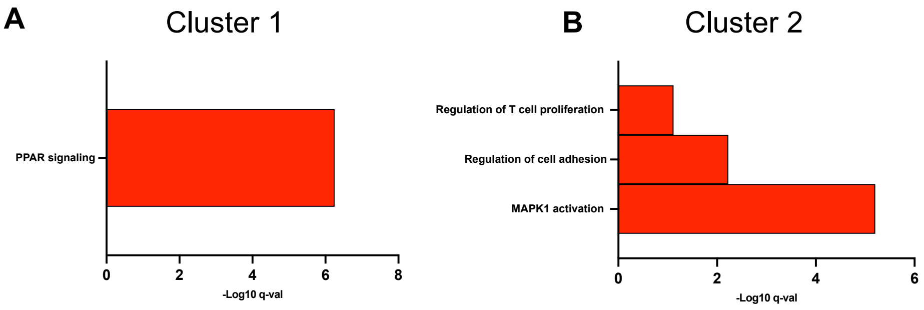

Fig. S4. Cluster analysis of PPARα signaling in liver of AFB1 exposed mice. (A) Cluster 1 and (B) Cluster 2.

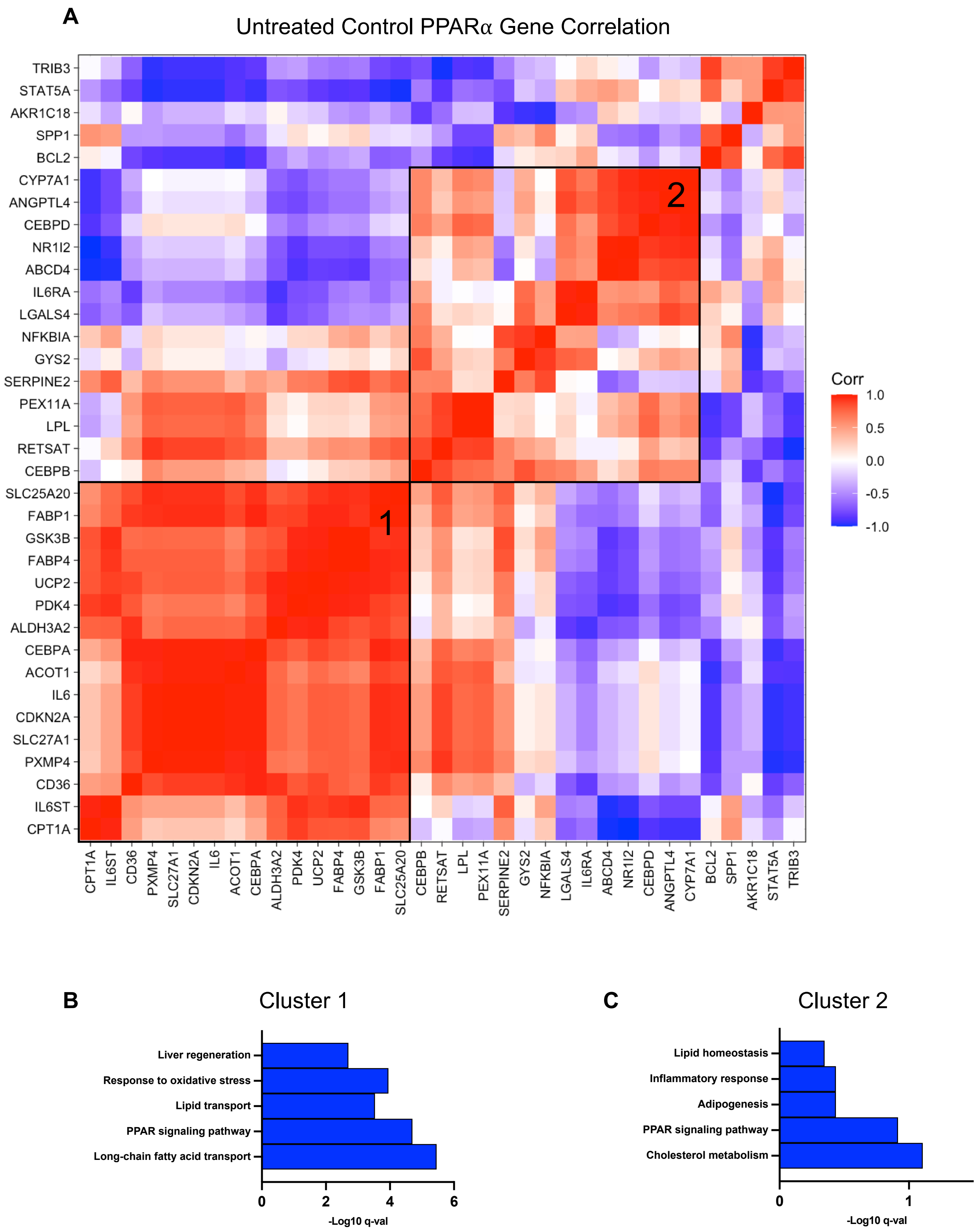

Fig. S5. PPARα gene correlation in liver of healthy control animals is remarkably like 10-HSA treated animals. (A) Pearson's correlation analysis plot of known PPARα regulated genes. Pathway analysis of (B) Cluster 1 and (C) Cluster 2.

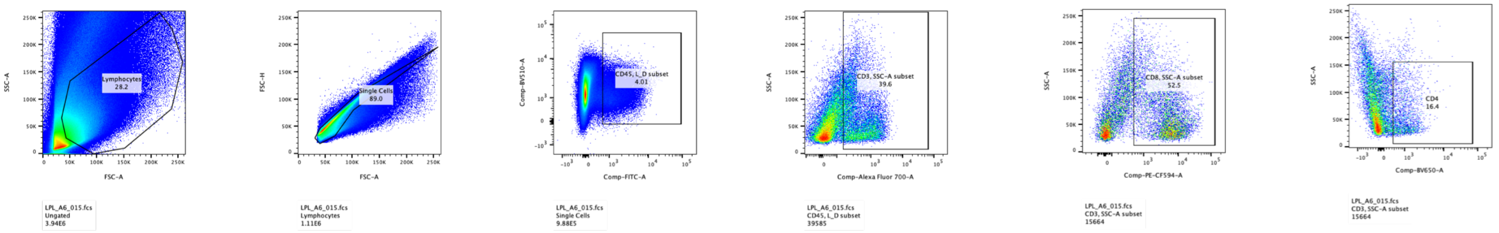

Fig. S6. Gating strategy for lamina propria T cells.

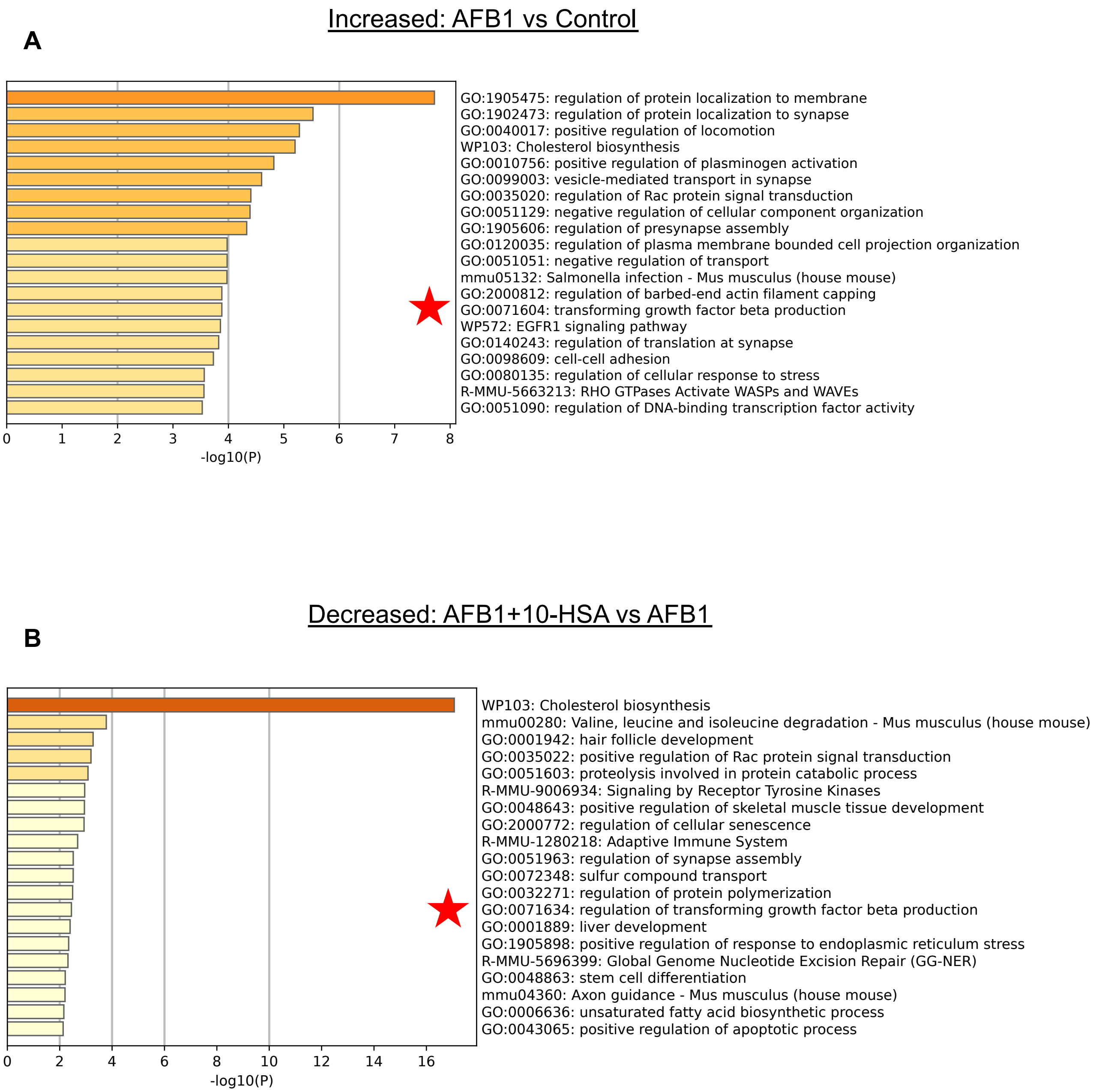

Fig. S7. AFB1 exposure increased TGF-β production and signaling in the gut. (A) Pathways from significantly increased gene expression in AFB1 compared to control group. (B) Pathways from significantly decreased gene expression in AFB1+10-HSA compared to AFB1 group.

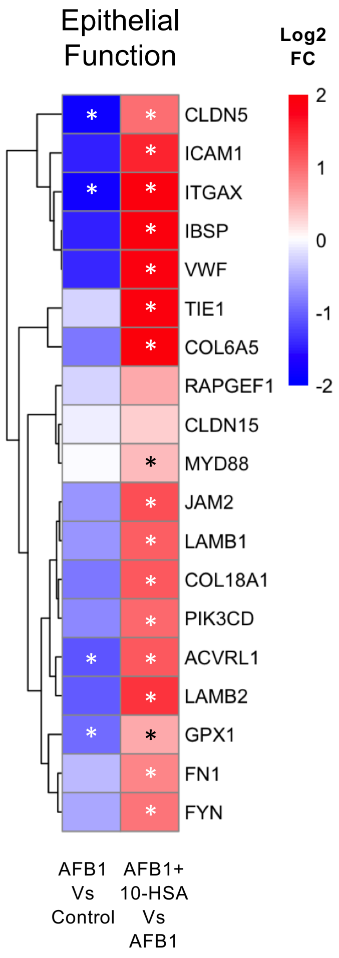

Fig. S8. Protection of gut epithelial integrity and function during AFB1 exposure.

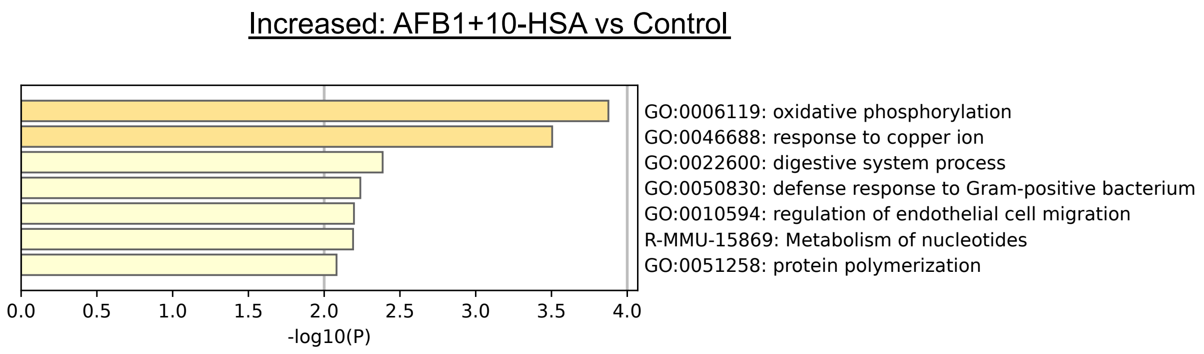

Fig. S9. 10-HSA in AFB1 exposed animals had significant increase of oxidative phosphorylation and energy metabolism expression pathways over untreated healthy controls.

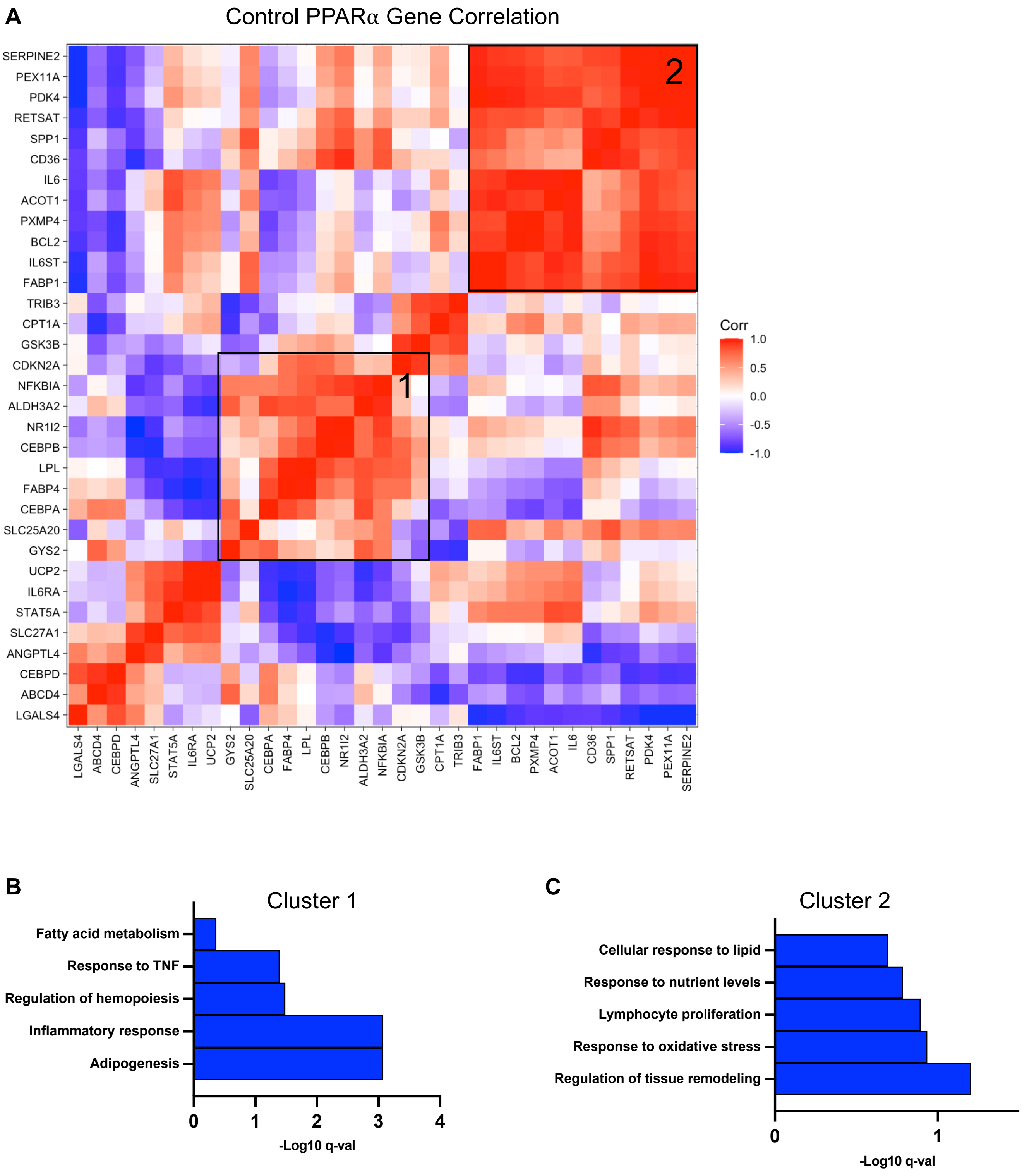

Fig. S10. PPARα gene correlation in gut of healthy control animals is remarkably like 10-HSA treated animals. (A) Pearson's correlation analysis plot of known PPARα regulated genes. Pathway analysis of (B) Cluster 1 and (C) Cluster 2.

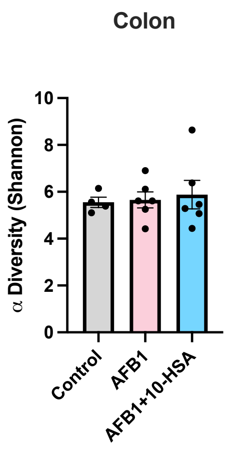

Fig. S11. Alpha diversity in colon.

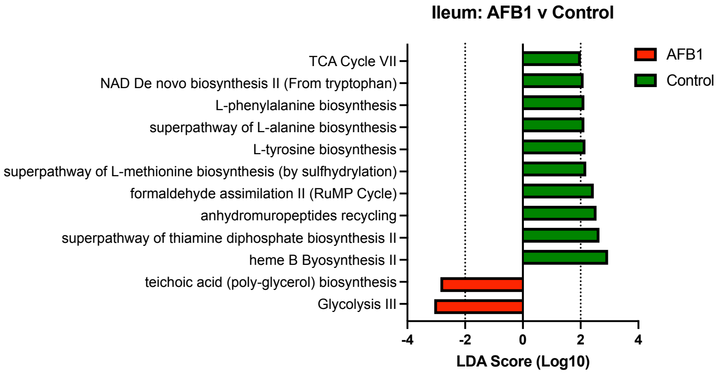

Fig. S12. AFB1 exposure suppressed gut microbiome amino-acid metabolism compared to healthy controls.

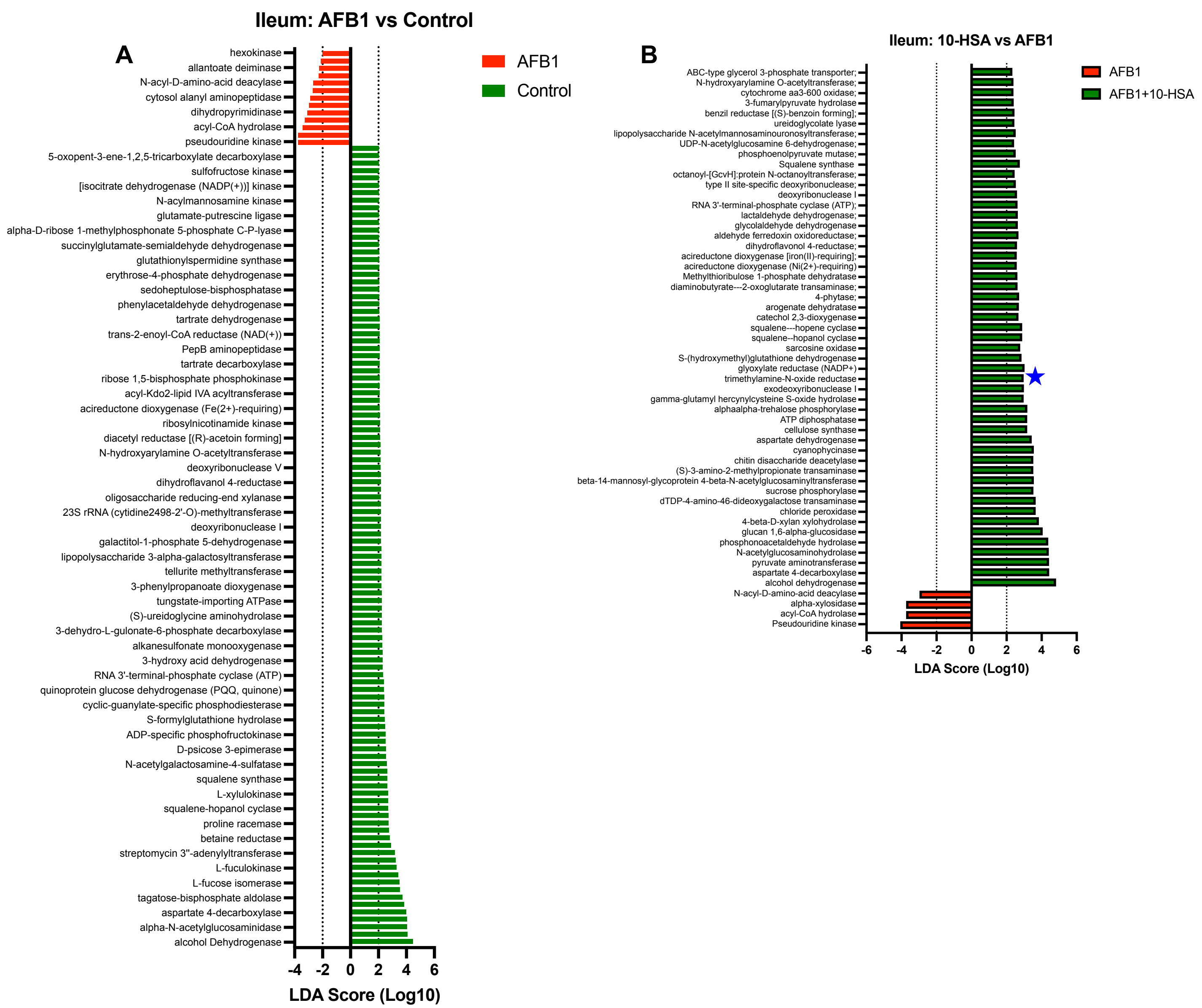

Fig. S13. AFB1+10-HSA animals had enhanced diversity of predicted bacterial enzymes in gut microbiomes compared to AFB1 exposed mice. (A) AFB1 vs healthy control LEfSe analysis of PICRUSt2 enzyme counts. (B) AFB1+10-HSA vs AFB1 LEfSe analysis of PICRUSt2 enzyme counts.

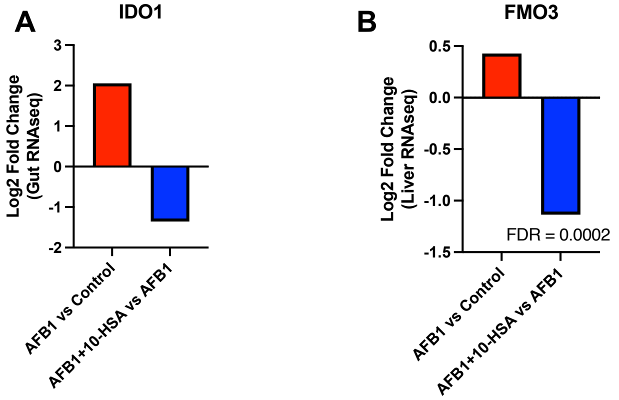

Fig. S14. 10-HSA reduced IDO1 expression in the gut and FMO3 expression in the liver. (A) IDO1 expression from gut RNAseq data. (B) FMO3 expression from liver RNAseq data.
